# Aspergillus fumigatus Drives Tissue Damage via Iterative Assaults upon Mucosal Integrity and Immune Homeostasis

**DOI:** 10.1128/iai.00333-22

**Published:** 2023-01-10

**Authors:** Uju Joy Okaa, Margherita Bertuzzi, Rachael Fortune-Grant, Darren D. Thomson, David L. Moyes, Julian R. Naglik, Elaine Bignell

**Affiliations:** a Centre for Host-Microbiome Interactions, Faculty of Dentistry, Oral & Craniofacial Sciences, King’s College London, London, United Kingdom; b Manchester Fungal Infection Group, Faculty of Biology, Medicine and Health, University of Manchester, Manchester, United Kingdom; c MRC Centre for Medical Mycology, University of Exeter, Exeter, United Kingdom; Tulane School of Medicine

**Keywords:** *Aspergillus fumigatus*, fungal infection, lung infection, virulence

## Abstract

The human lung is constantly exposed to Aspergillus fumigatus spores, the most prevalent worldwide cause of fungal respiratory disease. Pulmonary tissue damage is a unifying feature of Aspergillus-related diseases; however, the mechanistic basis of damage is not understood. In the lungs of susceptible hosts, A. fumigatus undergoes an obligatory morphological switch involving spore germination and hyphal growth. We modeled A. fumigatus infection in cultured A549 human pneumocytes, capturing the phosphoactivation status of five host signaling pathways, nuclear translocation and DNA binding of eight host transcription factors, and expression of nine host response proteins over six time points encompassing exposures to live fungus and the secretome thereof. The resulting data set, comprised of more than 1,000 data points, reveals that pneumocytes mount differential responses to A. fumigatus spores, hyphae, and soluble secreted products via the NF-κB, JNK, and JNK + p38 pathways, respectively. Importantly, via selective degradation of host proinflammatory (IL-6 and IL-8) cytokines and growth factors (FGF-2), fungal secreted products reorchestrate the host response to fungal challenge as well as driving multiparameter epithelial damage, culminating in cytolysis. Dysregulation of NF-κB signaling, involving sequential stimulation of canonical and noncanonical signaling, was identified as a significant feature of host damage both in vitro and in a mouse model of invasive aspergillosis. Our data demonstrate that composite tissue damage results from iterative (repeated) exposures to different fungal morphotypes and secreted products and suggest that modulation of host responses to fungal challenge might represent a unified strategy for therapeutic control of pathologically distinct types of Aspergillus-related disease.

## INTRODUCTION

Aspergillus fumigatus is an opportunistic fungal pathogen and a major cause of human lung disease. A. fumigatus spores are common among the airborne microflora and are associated annually with ~200,000 human fatalities, occurring in severely immunocompromised patients, including patients with organ or stem cell transplants, HIV/AIDS, or underlying chronic pulmonary disease (1). In asthma, bronchiectasis, and cystic fibrosis settings, inhalation of A. fumigatus spores also causes millions of debilitating respiratory malfunctions, including allergic bronchopulmonary aspergillosis and chronic pulmonary aspergillosis (1). With the emergence of new risk factors such as severe acute respiratory syndrome coronavirus 2, chimeric antigen receptor T-cell therapy, and intensive care unit stay, Aspergillus-related morbidity and mortality remain increasingly high (2).

During contact with the pathogen, airway epithelial cells (AECs) are iteratively exposed to A. fumigatus dormant spores, swollen spores, elongated hyphae, and secreted fungal products, all of which elicit host responses that can culminate in diverse disease pathologies (3).

In both oral and respiratory epithelial cells, MAPK signaling through ERK/JNK/p38 kinases has been shown to play important roles in responses to fungal challenges (4). In human airway epithelial cells, MAP kinases may regulate downstream gene expression by NF-κB-dependent and -independent processes, as well as by posttranscriptional mechanisms. Previous studies demonstrated a cross-talk between JNK signaling activation and NF-κB activation, whereby JNK activation reduced the phosphorylation and subsequent degradation of IκB-α, which is required for canonical NF-κB transcriptional responses (5). Both classical and alternative NF-κB activation pathways have been shown to contribute to airway allergic inflammation and remodeling in response to house dust mite challenge (6).

In addition to the physical invasion of lung tissues, A. fumigatus pathogenicity is also driven by secreted factors produced by the fungus, such as proteases and immunotoxins, which become expressed downstream of an obligatory morphogenetic switch from a spore to a hyphal form (7–12). The iterative interactions of A. fumigatus spore and hyphal forms with host cells are integral features of the host-pathogen interaction; however, our current understanding of these interactions has been compiled from disparate, mostly single time point studies that have used different types of AECs and various A. fumigatus morphotypes (11). In their study, Zhang et al. (13) showed that both heat-inactivated and irradiated A. fumigatus mycelium induced the production of interleukin-8 (IL-8) at a single time point of 24 h. Subsequently, another study investigated the early transcriptional response of epithelial cell lines in response to germinating conidia (14), reporting a significant increase in mRNA levels of key (IL-6, IL-8, and tumor necrosis factor-α [TNF-α]) cytokine-encoding genes from 8 h to 24 h of interaction with fungal spores, corresponding to the timescale of germination and hyphal production. An increase in IL-8 encoding gene expression was observed in response to the conidial challenge from 8 h postinfection and was dependent on MAPK p38 and ERK1/2 pathways (13). No studies have yet reported dynamic, multiparameter analysis of epithelial integrity, host signaling activation, nuclear translocation of transcription factors, and resultant effector expression in response to A. fumigatus *challenge.* Among other fungal pathogens that colonize and infect host mucosa, evidence of morphotype-specific activation of host responses has been demonstrated in response to Candida albicans challenge (7, 8). Hypothesizing that sequential, morphotype-specific host responses to A. fumigatus challenge drive cumulative damage during A. fumigatus infection, we sought to capture the dynamic epithelial responses to A. fumigatus as a means to study the relative contributions of pathogen and host activities to epithelial damage.

## RESULTS

### Aspergillus fumigatus spores, hyphae, and secreted products drive epithelial decay via temporally and mechanistically distinct processes.

**FIG 1:**
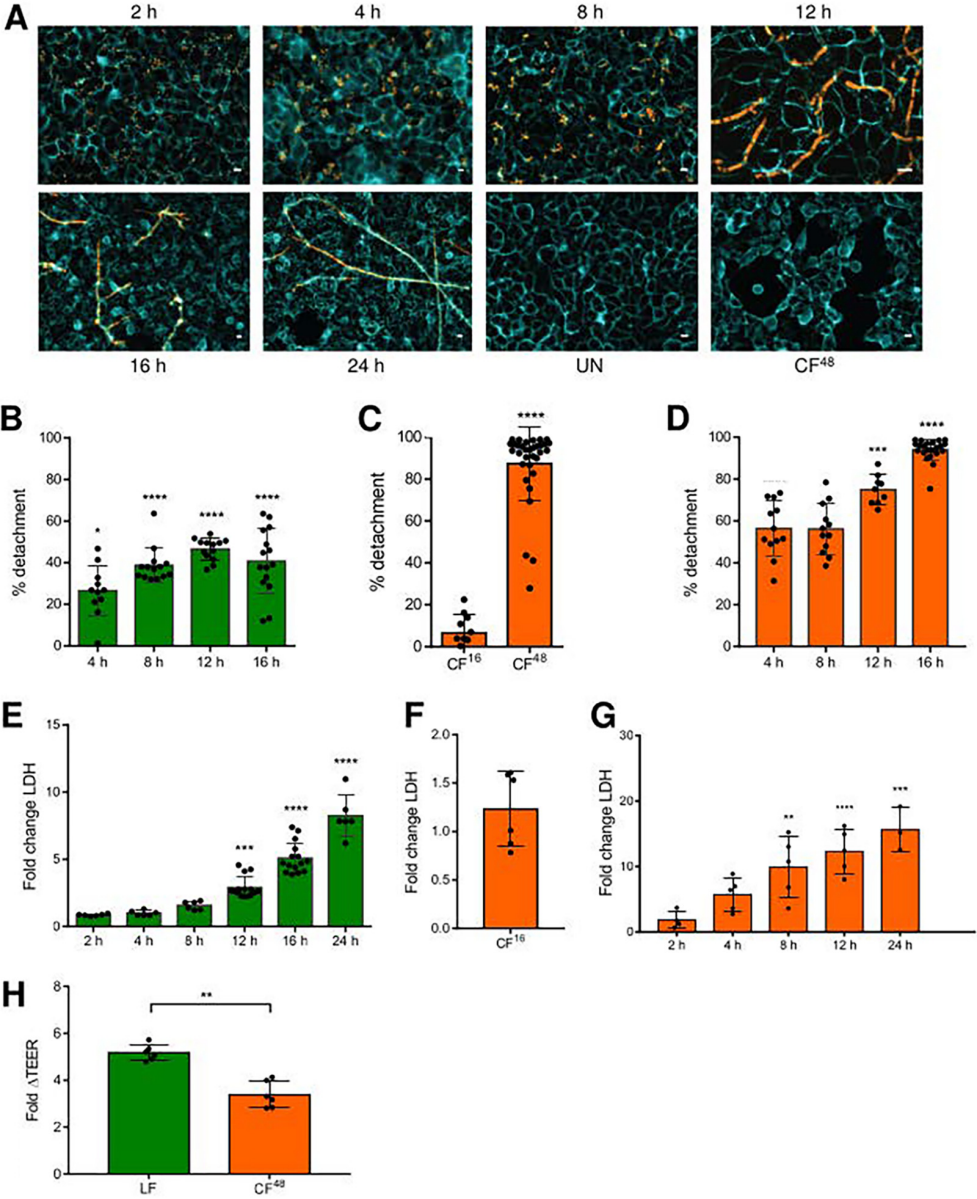
Temporal quantitative analysis of epithelial decay following challenge with live A. fumigatus spores (LF) or CF. (A) Visualization of concanavalin A-FITC-labeled A549 monolayers incubated with the A. fumigatus tdTomato^ATCC4664^ strain (MOI = 1) and a 5-fold diluted culture filtrate thereof (CF^48^). (B) Percentage of detachment of A549 cells following infection with 10^6^ A. fumigatus CEA10 spores at indicated time points. (C) Percentage of detachment of A549 cells following 16 h challenge with a 5-fold diluted filtrate from CEA10 fungal cultures (inoculum of 10^6^ spores/mL) grown for 16 h (CF^16^) or 48 h (CF^48^). (D) Percentage of detachment of A549 cells following challenge with a 5-fold diluted filtrate from CEA10 fungal cultures at indicated time points. (E) Fold change LDH release (relative to PBS challenge) at indicated time points with 10^6^ A. fumigatus CEA10 spores. (F) Fold change LDH release (relative to PBS challenge) at 24 h postexposure to CF^16^. (G) Fold change LDH release (relative to PBS challenge) at indicated time points with a 5-fold diluted filtrate from CEA10 fungal cultures. (H) Fold change decrease in TEER between A549 monolayers incubated for 24 and 0 h with A. fumigatus CEA10 spores and a 5-fold diluted filtrate from CEA10 fungal cultures. Data represent 3 biological replicates with 1 to 5 technical replicates. Error bars show ± SEM. Data were analyzed by nonparametric one-way ANOVA (Kruskal-Wallis test) with Dunn’s multiple comparisons. Significance was calculated relative to challenge with vehicle control (PBS) unless otherwise shown by brackets. ****, *P* ≤ 0.0001; ***, *P* ≤ 0.001; **, *P* ≤ 0.01; and *, *P* ≤ 0.05.

A. fumigatus causes epithelial detachment and damage via multiple, temporally distinct mechanisms (3). To define the dynamics of the underlying processes and address the role of host cell activities in driving epithelial damage, A549 cells were challenged with live A. fumigatus spores or culture filtrates (CFs). Wide-field fluorescence microscopy was used to study the gross appearance of epithelial monolayers at set time points postinfection (Fig. 1A) and to quantify the degree of AEC detachment (Fig. 1B to D and see Fig. S1B in the supplemental material).

Germination of A. fumigatus was observed at approximately 8 h, followed by a phase of rapid hyphal extension between 8 and 12 h postinfection (Fig. 1A). In response to challenge with live A. fumigatus spores, significant detachment (~20%) of A549 cells was detected by 4 h (Fig. 1B), when the fungus is still a spore (Fig. 1A and B). Upon spore germination (8 to 12 h), pneumocyte detachment further increases to reach 40 to 50% (Fig. 1B). To assess the role of secreted fungal products in driving epithelial decay, AEC detachment was quantified following exposure for 16 h to CF harvested from fungal broth culture at 16 (CF^16^) or 48 (CF^48^) h. Pneumocyte detachment was caused by exposure to CF^48^ but not to CF^16^. Therefore, the cytolytic capacity of A. fumigatus increases >80-fold within the 16 to 48 h time frame (Fig. 1C). In contrast to live fungal spores (Fig. 1B), exposure to CF^48^ led to much more rapid epithelial decay (Fig. 1D). The magnitude of epithelial disintegration in response to challenge with spores and CF^48^ was independent of the A. fumigatus strain tested (Fig. S1B).

To determine whether epithelial detachment was caused by cytolytic death of AECs, lactate dehydrogenase (LDH) release was quantified over a similar time course of fungal challenge. In response to challenge with live A. fumigatus spores, LDH release first significantly exceeded that due to PBS challenge at 12 h postinfection (Fig. 1E), which correlated with the onset of hyphal development (Fig. 1A). LDH activity increased in a dose-dependent manner proportionate to increasing spore density of the fungal inoculum (Fig. S1C). In contrast to spore exposure where cytolytic damage takes 12 h to become detectable, AECs challenged with CF^48^ exhibited a rapid time- and dose-dependent lysis, which commenced at 2 h postchallenge (Fig. 1G and Fig. S1D). Notably, as with epithelial detachment, CF^16^ did not induce LDH release (Fig. 1F). The magnitude of epithelial cell lysis in response to challenge with spores and CF^48^ was independent of the A. fumigatus strain tested although timescales differed slightly (Fig. S1E).

Due to their physiology, Calu-3 cell models are regarded as useful for the study of tight junction integrity. To quantify the relative contributions of living fungal cells versus hypha-derived soluble factors in driving tight junction decay, the integrity of Calu-3 monolayers was measured by monitoring changes in transepithelial resistance (TEER) at 24 h following challenge with live A. fumigatus spores or CF^48^ (Fig. 1H and S1F). Infection of monolayers with live fungal spores for 24 h reduced TEER by 5-fold relative to an uninfected monolayer (Fig. 1H), whereas exposure to CF^48^ for 24 h reduced TEER by only 2 to 3-fold relative to an uninfected monolayer (Fig. 1H). The decrease in TEER in response to challenge with spores and CF^48^ was independent of the A. fumigatus strain tested (Fig. S1G). Taken together these findings suggest that fungal spores are involved in initiating epithelial damage, which is then further propagated by (i) physical invasion of the epithelial layer and (ii) effector-mediated lysis of epithelial cells.

### Cytokine and extracellular matrix responses to A. fumigatus spores are subsequently remodeled by secreted fungal products.

**FIG 2:**
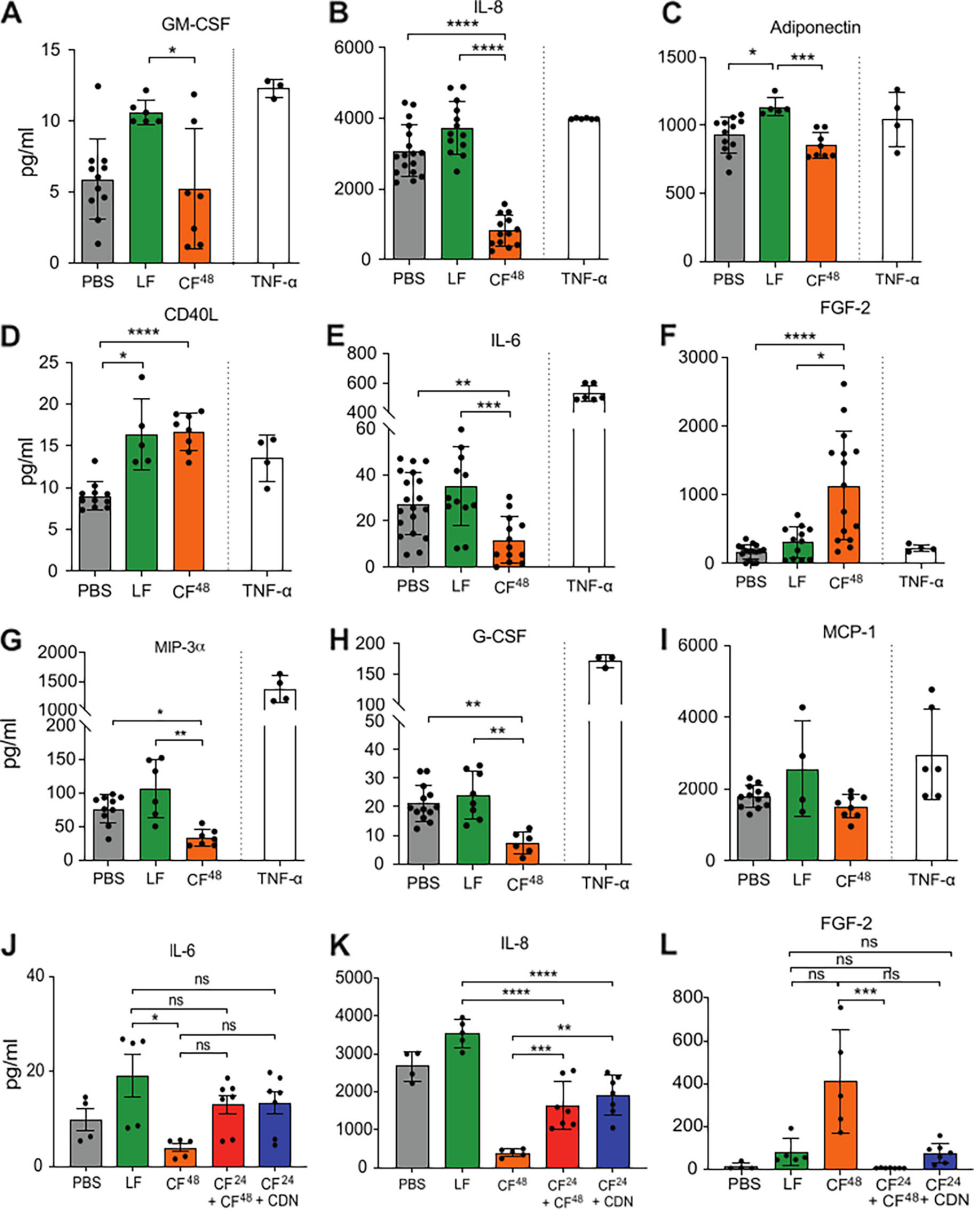
Differential cytokine secretion by A549 cells in response to live A. fumigatus spores (LF) and CF^48^. (A to I) Secreted cytokines GM-CSF (A), IL-8 (B), adiponectin (C), CD40L (D), IL-6 (E), FGF-2 (F), MIP-3a (G), G-CSF (H), and monocyte chemoattractant protein-1 (MCP-1) (I) were quantified in cell culture supernatant following exposure to A. fumigatus spores (1 × 10^7^ spores/mL) (MOI = 10) or 5-fold diluted CF^48^ for 24 h. (J to L) Cytokines IL-6 (J), IL-8 (K), and FGF-2 (L) were quantified in A549-free culture supernatants collected after exposure of epithelial monolayers to A. fumigatus for 24 h (CF^24^) followed by incubation with CF^48^ or with culture filtrates recovered from a 24 h infection of AECs with CF^48^ (CDN). Data represent 3 biological replicates with 1 to 5 technical replicates. Error bars show ± SEM. Data were analyzed by nonparametric one-way ANOVA (Kruskal-Wallis test) with Dunn’s multiple comparisons. Significance was calculated relative to challenge with vehicle control (PBS) and between each treatment as shown. ****, *P* ≤ 0.0001; ***, *P* ≤ 0.001; **, *P* ≤ 0.01; and *, *P* ≤ 0.05.

In addition to cytolytic damage resulting from direct host-pathogen interaction, the subversion of innate host defenses can also indirectly promote tissue damage via disablement or moderation of the protective host immune response. To characterize the effects of fungal challenge on host-derived cytokine and extracellular matrix (ECM) responses, AECs were exposed to live A. fumigatus spores or fungal secreted products (CF^48^) and expression of cytokines and (ECM) components were quantified via (i) an unbiased high-throughput immunoblotting approach (Data set S4) and (ii) targeted quantitation of nine host proteins exhibiting significant changes, including IL-6 and IL-8, which have been previously reported to be induced by A. fumigatus in bronchial and alveolar epithelial cells (13, 15) and granulocyte-macrophage colony-stimulating factor (GM-CSF), granulocyte colony-stimulating factor (G-CSF), IL-6, and IL-8 by C. albicans in a buccal epithelial carcinoma cell line (8) (Fig. 2).

AEC challenge with A. fumigatus spores for 24 h significantly increased the secretion of GM-CSF, IL-8, adiponectin, and CD40L, relative to the vehicle control (Fig. 2A to D). A nonsignificant increase in IL-6, FGF-2, and macrophage inflammatory protein-3 alpha (MIP-3a) secretion was also observed (Fig. 2E to G). In stark contrast, relative to challenge with live fungus, challenge with CF^48^ for 24 h reduced the concentrations of most cytokines to less than basal (PBS) levels. However, secretion of FGF-2 and CD40L was significantly increased in response to CF^48^ challenge (Fig. 2D and F). The reductions in IL-6 and IL-8 secretion might have derived from rapid epithelial decay or direct action of fungal proteases that degrade cytokines. To distinguish between the two possibilities, conditioned media harvested from epithelial culture at 24 h postinfection with live fungus (CDN^24^), and therefore exhibiting heightened cytokine concentrations, were subsequently exposed to culture filtrates obtained from mature fungal cultures grown in the presence (CDN^48^) or absence (CF^48^) of host cells. This revealed that IL-6 depletion in response to CDN^24^ challenge does not significantly differ from challenge with live fungus, thereby negating a role for fungal proteases in IL-6 degradation. Conversely, IL-8 is significantly depleted by exposure to CDN^24^ causing an ~50% reduction in IL-8 concentration relative to challenge with live fungus (Fig. 2J to K). Epithelial challenge with CF^48^ prompted significantly increased FGF-2 secretion, an effect that could not be recapitulated via CDN^24^ challenge (Fig. 2F and L), thereby indicating that FGF-2 induction depends on the presence of one or multiple soluble factors produced by mature fungal hyphae. Taken together, these data indicate that A. fumigatus-secreted products act in a target-specific manner to remodel the local cytokine and extracellular matrix environment during epithelial infection.

### Spore germination in proximity to pneumocytes sequentially stimulates NF-κB and MAPK signaling and DNA binding of downstream transcription factors.

**FIG 3:**
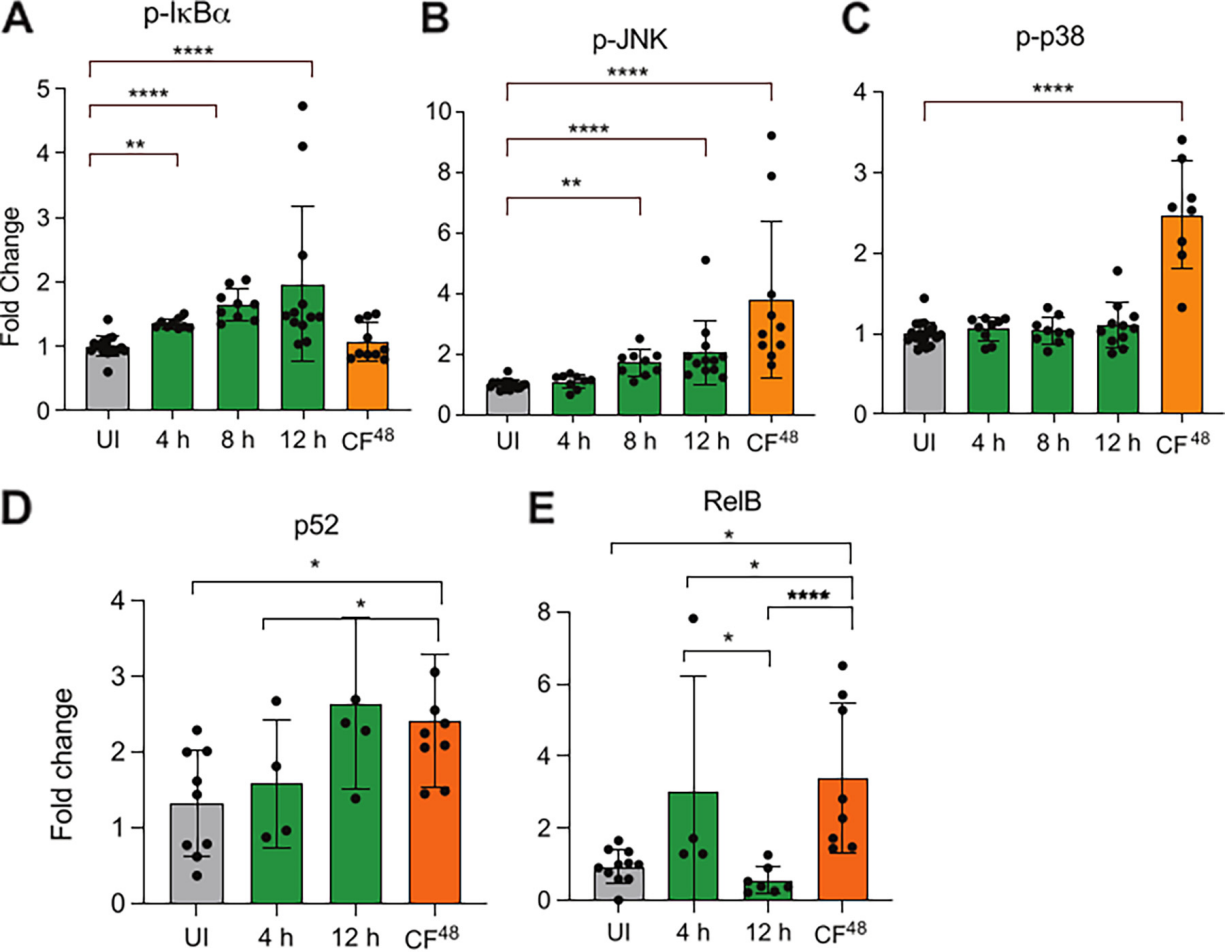
Different fungal morphotypes induce differential and dynamic host responses in A549 cells. (A to C) Fold change (relative to uninfected control [UI]) phosphorylation of NF-κB (p-IkBα [A]) and MAPK (p-JNK [B]and p-p38 [C]) signaling following exposure to A. fumigatus spores (1 × 10^7^ spores/mL) (MOI = 10) for indicated time points or 5-fold diluted CF^48^ for 4 h. (D to E) Fold change (relative to uninfected control, UI) in DNA binding activity of noncanonical NF-κB transcription factors (p52 [D] and RelB [E]) following exposure to A. fumigatus spores (10^7^ spores/mL) for indicated time points or 5-fold diluted CF^48^ (inoculum of 10^6^ spores/mL) for 4 h. Data represent 3 biological replicates with 1 to 5 technical replicates. Error bars show ± SEM. Data were analyzed by nonparametric one-way ANOVA (Kruskal-Wallis test) with Dunn’s multiple comparisons. Significance was calculated relative to challenge with vehicle control (PBS) as shown. ****, *P* ≤ 0.0001; ***, *P* ≤ 0.001; **, *P* ≤ 0.01; and *, *P* ≤ 0.05.

Although the ability of AECs to orchestrate immunological responses in response to A. fumigatus has been previously documented (15–18), the fungal morphotypes propagating intracellular signaling have remained obscure. The phosphorylation status of three mitogen-activated protein kinases (MAPKs) (p38, ERK, and JNK), the inhibitor of kappa-light-chain-enhancer of activated B cells (IκBα) and of protein kinase B (AKT), and nuclear translocation of downstream transcription factors (p52, p50, RelB, p65, MEF-1, c-Myc, and JunD), including several known to be responsive to C. albicans challenge of oral epithelial cells or keratinocytes (8, 17, 19, 20), was measured over a time series of coincubation with live A. fumigatus spores or CF^48^. Unlike the canonical NF-κB signaling, activation of noncanonical NF-κB transcription factors occurs independently of NEMO/IκB-α complex and exclusively via activation of the NF-κB-inducing kinase (NIK), which phosphorylates IKKα. Phosphorylation of p100 by IKKα triggers partial degradation to p52, which is capable of forming transcriptionally active RelB/p52 dimers of the noncanonical pathway (21). The earliest evidence of a host response to fungal spore challenge was observed from 4 h postinfection at which time a modest, but significant, increase in IκB-α phosphorylation (Fig. 3A) was observed that persisted throughout the course of infection. A similarly modest phosphorylation of JNK (Fig. 3B) was observed. Phosphorylation of p38 (Fig. 3C), p-Akt, and p-ERK1/2 (Fig. S2E) did not occur in response to challenge with spores.

Exposure of A549 cells to CF^48^ led to an immediate increase in phosphorylation (3- and 1.5-fold respectively, *P* < 0.0001) of JNK and p38 at 4 h postexposure (Fig. 3B and C). In contrast, phosphorylation of IkBα, a key event in NF-κB signaling activation, was not observed during challenge with CF^48^ (Fig. 3A), suggesting that NF-κB activation in response to A. fumigatus challenge is most likely contact mediated. The magnitude of IκB-α, JNK, and p38 phosphorylation in response to challenge with spores was independent of the A. fumigatus strain tested (Fig. S2A to C). However, in line with an expanding number of studies showing strain to strain variation of A. fumigatus phenotypes, immune responses, and virulence in various models of infection (22), CF^48^ from the three parental isolates tested (CEA10, ATCC 46645, and a nonhomologous end joining deficient mutant *ΔakuB*^KU80^) elicited JNK and p38 phosphorylation to significantly different extents (Fig. S2A to C). In our experimental setup, no changes in Akt or ERK phosphorylation were observed following challenge with CF^48^ (Fig. S2D to E).

Activation of distinct signaling mechanisms by live spore challenge and CF^48^ was paralleled by differential modulation of DNA binding of associated transcription factors (Fig. 3D and E and Fig. S4). Upon epithelial challenge with live A. fumigatus for 12 h, DNA binding of Jun D and MEF-2 decreased significantly (0.5-fold, *P* < 0.05 and *P* < 0.01, respectively), while DNA binding of c-Myc increased (1.75-fold, *P* < 0.05) (Fig. S3A to C).

Importantly, CF^48^ challenge of epithelial monolayers for 4 h led to a significant increase in DNA binding (1- and 3-fold, respectively, *P* < 0.05) of the p52 and RelB components of noncanonical NF-κB signaling (Fig. 3D and E). No significant changes in ATF-2, c-Jun, FosB, c-Fos, Fra1, c-Rel, STAT-1, JunB (not shown), p50 (Fig. S3D), or p65 (Fig. S3E) binding activity were observed in our population-level assays. However, extensive variation of the experimental readings for these transcription factors was observed. This might be indicative of heterogeneity among the cell population studied and/or rapid cycling between phosphorylated and unphosphorylated states of activity in response to fungal challenge.

### A. fumigatus-induced epithelial damage is mediated by IκB-α and p-JNK activation.

**FIG 4:**
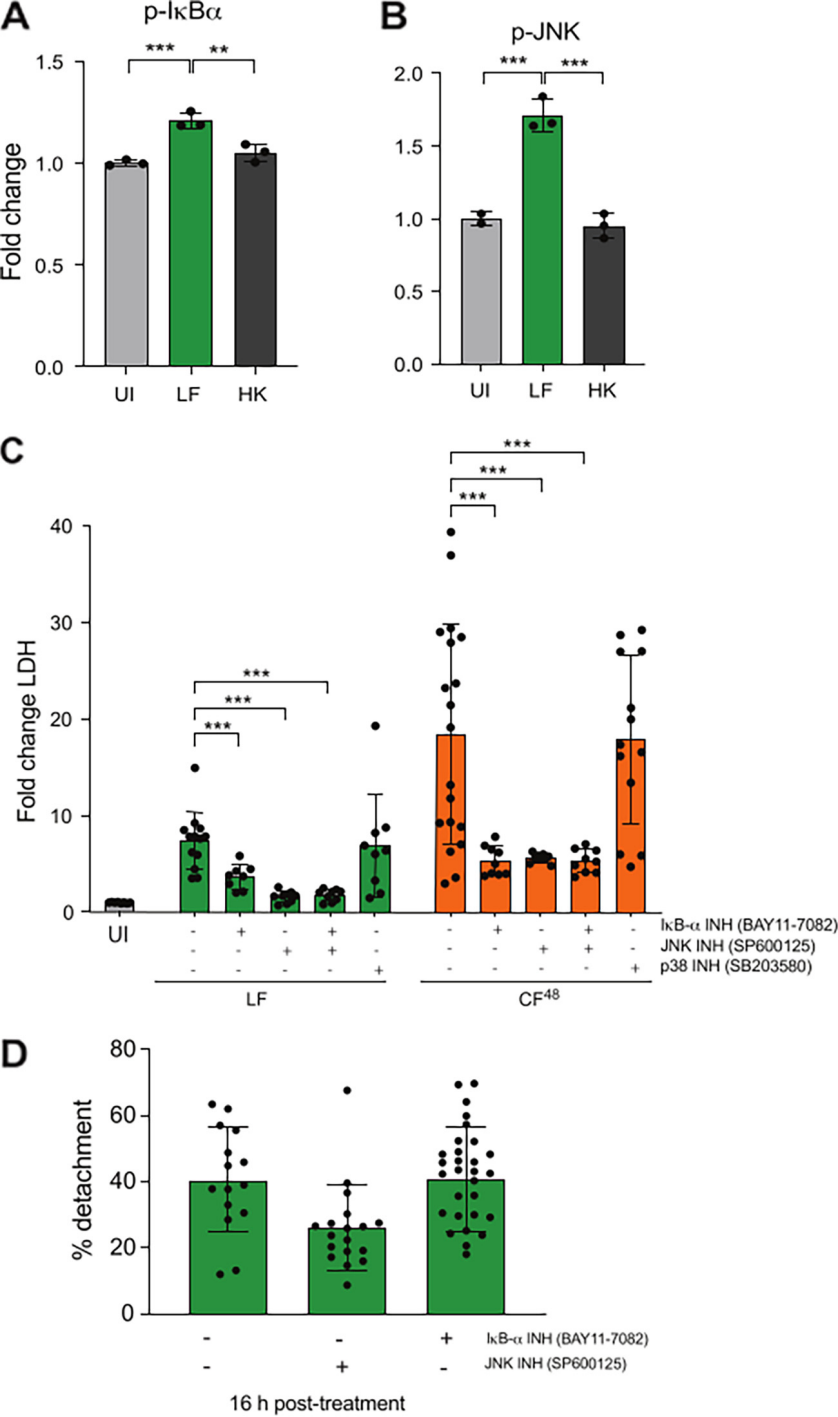
Signaling activation is dependent on fungal viability and plays a role in epithelial damage. (A and B) Fold change (relative to uninfected control [UI]) phosphorylation of NF-κB (p-IkBα [A]) and MAPK (p-JNK [B]) signaling following 4 h exposure to live fungus (LF) and heat-killed (HK) morphotypes (10^7^ spores/mL) of A. fumigatus pregrown to hyphae for 12 h (MOI = 10). (C) Fold change LDH release (relative to PBS challenge) following chemical inhibition of NF-κB (IkBα inhibitor BAY11-7082) and MAPK (JNK inhibitor SP600125 and p38 inhibitor SB203580) signaling pathways and exposure to A. fumigatus spores (10^6^ spores/mL) MOI = 1- or 5-fold diluted CF^48^ for 24 h. (D) Fold change detachment (relative to PBS challenge) following chemical inhibition of NF-κB (IkBα inhibitor BAY11-7082) and MAPK (JNK inhibitor SP600125) signaling pathways and exposure to A. fumigatus spores (10^6^ spores/mL) MOI = 1 for 16 h. Data represent 2 or 3 biological replicates with 1 to 5 technical replicates. Error bars show ± SEM. Data were analyzed by nonparametric one-way ANOVA (Kruskal-Wallis test) with Dunn’s multiple comparisons. Significance was calculated relative to challenge with vehicle control (PBS) and between each treatment as shown. ****, *P* ≤ 0.0001; ***, *P* ≤ 0.001; **, *P* ≤ 0.01; and *, *P* ≤ 0.05.

Epithelial perturbation, detachment, and lysis are key features of A. fumigatus *in vitro* infections first in a contact-dependent manner and subsequently via the secretion of fungal soluble factors (Fig. 1) (3). Notably, increased phosphorylation of both IκB-α and JNK is observed at 12 h postinfection with live fungus (Fig. 3A and B), thereby suggesting that hyphal-dependent activation of the NF-κB and MAPK pathways leads to A. fumigatus-induced epithelial damage. To determine if phosphorylation of IκB-α and JNK requires live fungus, A. fumigatus was grown for 12 h to induce hyphae formation and A549 AECs were challenged with pregrown heat-killed and live A. fumigatus. Contrary to live A. fumigatus pregrown as hyphae (up to 1.75-fold increase for IκB-α and JNK relative to uninfected, *P* ≤ 0.001), the heat-killed morphotypes failed to induce p-IκB-α and p-JNK activation upon challenge of epithelial monolayers, indicating that fungal viability is a prerequisite for phosphorylation of IκB-α and JNK (Fig. 4A and B).

The relative contribution of NF-κB and MAPK pathways in AEC lysis was determined using small chemical inhibitors against IκB-α (BAY11-7082), JNK (SP600125), and p38 (SB203580) (Fig. 4C). The p38 inhibition did not impact epithelial damage upon challenge with live fungus or CF^48^; however, JNK inhibition significantly reduced hyphal- and CF^48^-induced epithelial lysis by over 60%, while IκB-α inhibition yielded a less pronounced protective effect for hyphal-induced lysis but a similar reduction in CF^48^ induced AEC lysis. No evidence of an additive or synergistic effect was observed when both IκB-α and JNK pathways were inhibited simultaneously. Analysis of epithelial cell detachment in response to live fungal challenge revealed that JNK but not IκB-α inhibition leads to a significant reduction in epithelial cell detachment at 16 h (Fig. 4D). Taken together, these results indicate that composite A. fumigatus-induced epithelial damage is mediated by IκB-α and JNK, but not p38 activation.

### JNK-mediated epithelial lysis requires A. fumigatus PacC, GliP, and PrtT.

Functional genomic studies have demonstrated that the pH-sensing regulator PacC governs *in vitro* epithelial damage and A. fumigatus pathogenicity in leukopenic mice, by modulating the expression of over 250 secreted proteins, in addition to the secondary metabolite gliotoxin and cell wall-associated proteins (3). However, deletion alone of the conserved positive regulator of secreted proteases PrtT (23, 24) or of the nonribosomal peptide synthetase GliP, essential for gliotoxin production (25–27), does not diminish the survival of leukopenic mice following A. fumigatus challenge. Taken together, these findings suggest redundancy and possibly cooperative action of soluble effectors such as proteases and secondary metabolites in driving epithelial damage and A. fumigatus pathogenicity.

**FIG 5:**
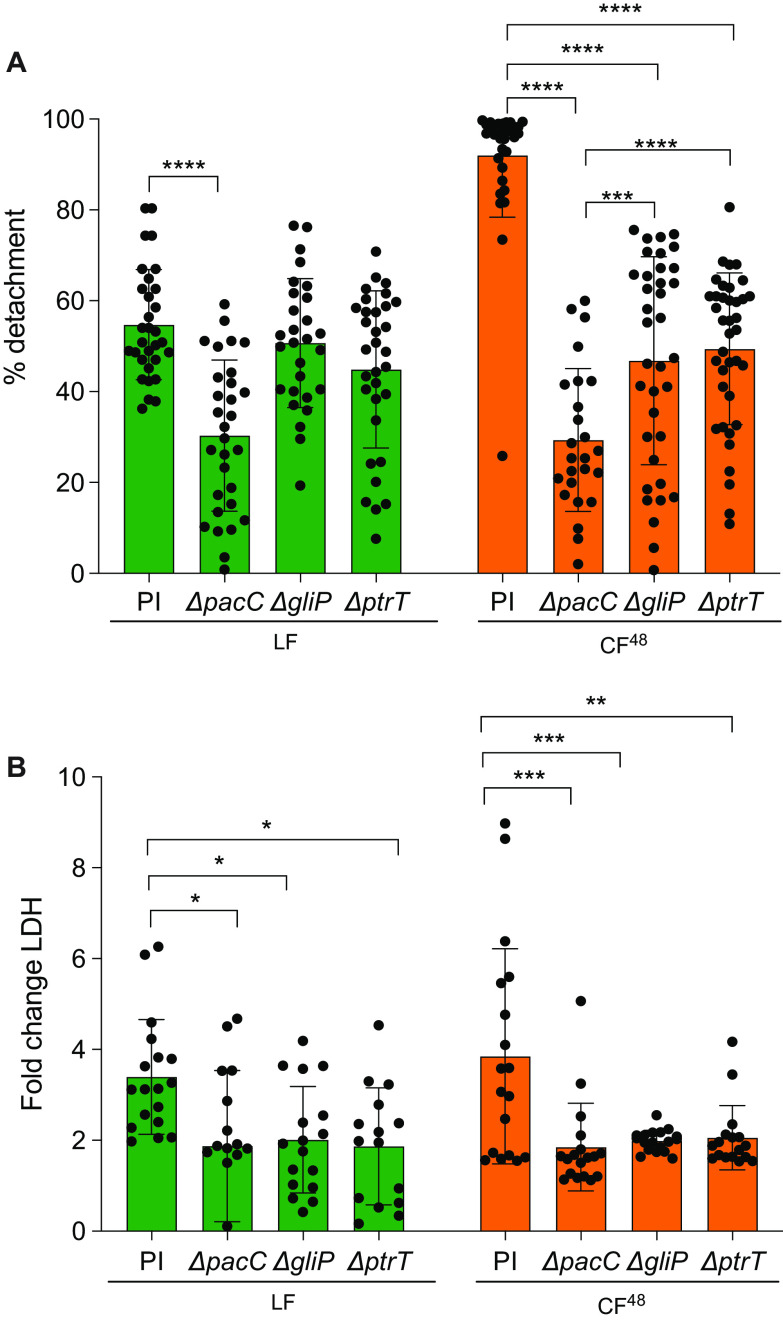
A. fumigatus Δ*pacC*, Δ*gliP,* and Δ*prtT* mutants and respective CF^48^ fail to induce AEC detachment and lysis. (A) Percentage of detachment of A549 cells following infection with 10^6^ A. fumigatus *ΔpacC*, *ΔgliP,* and *ΔprtT* mutants and respective parental isolate (PI) and CF^48^ thereof for 16 h. (B) Fold change LDH release (relative to PBS challenge) following infection with 10^6^ A. fumigatus *ΔpacC*, *ΔgliP*, and *ΔprtT* mutants and respective parental isolate (PI) and CF^48^ thereof for 24 h. Data represent 3 biological replicates with 1 to 5 technical replicates. Error bars show ± SEM. Data were analyzed by nonparametric one-way ANOVA (Kruskal-Wallis test) with Dunn’s multiple comparisons. Significance was calculated relative to the parental isolate (PI) as shown. ****, *P* ≤ 0.0001; ***, *P* ≤ 0.001; **, *P* ≤ 0.01; and *, *P* ≤ 0.05.

**FIG 6:**
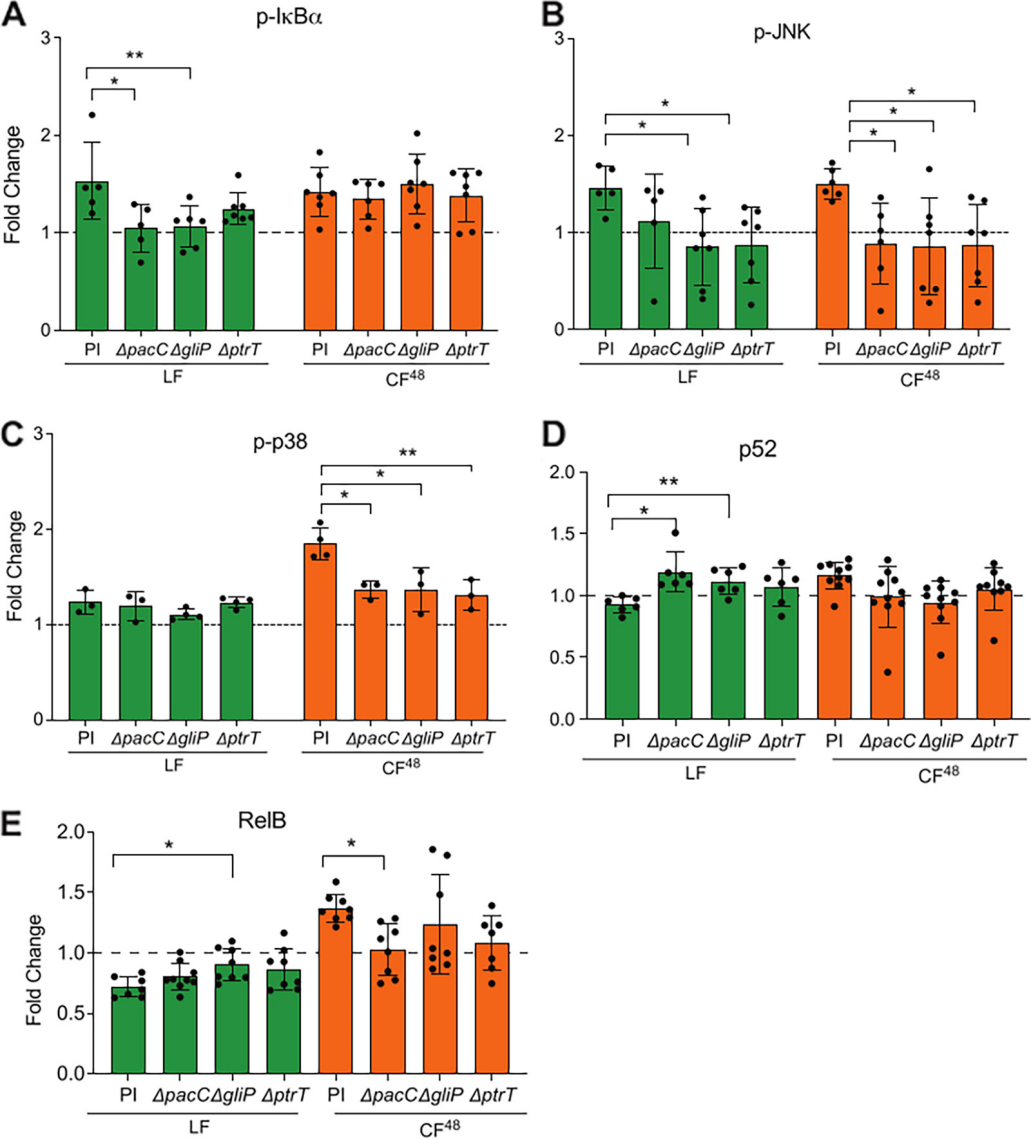
A. fumigatus Δ*pacC*, Δ*gliP,* and Δ*prtT* mutants and respective CF^48^ fail to activate AEC host signaling proteins and transcription factors. (A to C) Fold change (relative to PBS challenge) phosphorylation of NF-κB (p-IkBα [A]) and MAPK (p-JNK [B] and p-p38 [C]) signaling following exposure to A. fumigatus *ΔpacC*, *ΔgliP* and *ΔprtT* mutants and respective parental isolate (PI) (1 × 10^7^ spores/mL) for 8 h or respective 5-fold diluted CF^48^ for 4 h. (D to E) Fold change (relative to PBS challenge) in DNA binding activity of noncanonical NF-κB transcription factors (p52 [D] and RelB [E]) following exposure to A. fumigatus *ΔpacC*, *ΔgliP* and *ΔprtT* mutants and respective parental isolate (PI) (1 × 10^7^ spores/mL) for 8 h or respective 5-fold diluted CF^48^ for 4 h. Data represent 3 biological replicates with 1 to 5 technical replicates. Error bars show ± SEM. Data were analyzed by nonparametric one-way ANOVA (Kruskal-Wallis test) with Dunn’s multiple comparisons. Significance was calculated relative to the parental isolate (PI) as shown. ****, *P* ≤ 0.0001, ***, *P* ≤ 0.001; **, *P* ≤ 0.01; and *, *P* ≤ 0.05.

To determine the role of PacC, gliotoxin biosynthesis, and protease production in promoting epithelial detachment (Fig. 5A), lysis (Fig. 5B), and immune responses (Fig. 6), AEC monolayers were infected with an isogenic panel of A. fumigatus *ΔpacC*, *ΔgliP*, and *ΔprtT* mutants and respective CF^48^. In agreement with data previously published by Bertuzzi et al. (3), both live spores and CF^48^ of the *ΔpacC* mutant induce significantly less epithelial cell detachment (30% and 75% respectively) than the parental isolate (Fig. 5A). In contrast to *ΔpacC* challenges, CF^48^ but not live spores of the *ΔprtT* and *ΔgliP* mutants showed a decreased capacity to elicit epithelial cell detachment compared to the respective parental isolate (50% reduction, *P* ≤ 0.0001), while live fungal spores of the two mutants induced a similar level of cell detachment compared to their parental counterpart (Fig. 5A). Importantly, the attenuation of epithelial cell detachment caused by *ΔprtT* and *ΔgliP* CF^48^ was significantly less pronounced than the attenuation measured for *ΔpacC* CF^48^ (*P* ≤ 0.0001) (Fig. 5A), thereby supporting additivity or cooperativity of secreted proteases and gliotoxin in eliciting epithelial cell detachment at early time points of *in vitro* infection.

In contrast to the respective parental isolate, *ΔpacC*, *ΔgliP*, and *ΔprtT* mutants were less able to induce epithelial cell lysis (Fig. 5B). Accordingly, LDH release in response to challenge with *ΔpacC*, *ΔgliP,* and *ΔprtT* live mutants and respective CF^48^ was significantly reduced compared to challenges with the respective parental isolate (2 and 3-fold reduction for live fungus, *P* ≤ 0.05, and CF^48^, *P* ≤ 0.001, respectively).

Redundancy in GliP- and PrtT-mediated effects on epithelial responses was also observed at the level of signaling and transcriptional activation (Fig. 6). In comparison with live fungal challenge with their respective parental isolate, activation of IκBα phosphorylation was significantly reduced upon challenge of monolayers with live A. fumigatus mutants lacking PacC, GliP, or PrtT (Fig. 6A). However, while the *ΔprtT* mutant showed only a moderate decrease in the activation of IκBα phosphorylation after 8 h of *in vitro* infection with live spores, the *ΔpacC* and *ΔgliP* mutants completely failed (*P* ≤ 0.05 relative to parental isolate challenge) to elicit IκBα activation (Fig. 6A). Similarly to CF^48^ challenge with the parental strain, none of the mutants induced IκBα phosphorylation in epithelial cells upon challenge with the respective CF^48^ (Fig. 6A). While both live parental isolate and respective CF^48^ were able to induce JNK phosphorylation, *ΔpacC*, *ΔgliP*, and *ΔprtT* mutants and CF^48^ thereof failed (*P* ≤ 0.05 relative to parental isolate challenges) to activate JNK (Fig. 6B). As opposed to *ΔgliP* and *ΔprtT*, however, challenges with a live *ΔpacC* isolate only resulted in a moderate reduction in JNK activation after 8 h of *in vitro* infection (Fig. 6B). Neither the parental isolate nor the mutants induced p38 phosphorylation in epithelial cells upon challenge with live fungus but, contrary to CF^48^ challenge with the parental strain, CF^48^ from the *ΔpacC*, *ΔgliP*, and *ΔprtT* mutants failed to significantly elicit p38 activation (Fig. 6C).

Relative to parental isolate challenges, an increase in the DNA binding of p52 was observed in response to live fungal challenges with the *ΔpacC* and *ΔgliP* mutants (Fig. 6D) and a slight but significant increase in DNA binding of RelB in response to live fungal challenge with *ΔgliP* mutant. However, the DNA binding of p52 and RelB was significantly decreased in response to challenge with CF^48^ from *ΔgliP* and *ΔpacC* mutants, respectively. Taken together, these results highlight that PacC-mediated transcriptional regulation, gliotoxin biosynthesis, and PrtT-mediated protease production critically collaborate to differentially induce MAPK- and NF-κB-mediated signaling in epithelial cells in response to challenge with live spores and CF^48^, therefore ultimately impacting on epithelial detachment, lysis, and responses during A. fumigatus infections.

### Spore inhalation by leukopenic mice subverts NF-κB homeostasis.

Exposure of epithelial monolayers to A. fumigatus *in vitro* leads to the sequential activation of canonical and noncanonical NF-κB signaling and respective transcriptional events as shown by the significant phosphorylation of IκB-α at 4 to 12 h postinfection with live fungus (Fig. 3A) and by the significant increase in DNA binding of the p52 and RelB components of the NF-κB signaling pathway following CF^48^ challenge (Fig. 3D and E). Importantly, *in vitro*, activation of NF-κB signaling leads to A. fumigatus-induced epithelial damage, as demonstrated by the significant reduction of LDH release following IκB-α phosphorylation and proteolysis in monolayers challenged with live fungus and respective CF^48^ (Fig. 4C). Furthermore, the noninvasive and attenuated A. fumigatus mutant lacking the transcription factor PacC evokes muted canonical and noncanonical NF-κB activity compared to that induced by an isogenic parental isolate (Fig. 5 and 6), strongly implicating NF-κB in damage-inducing host responses to fungal challenge during infection.

**FIG 7:**
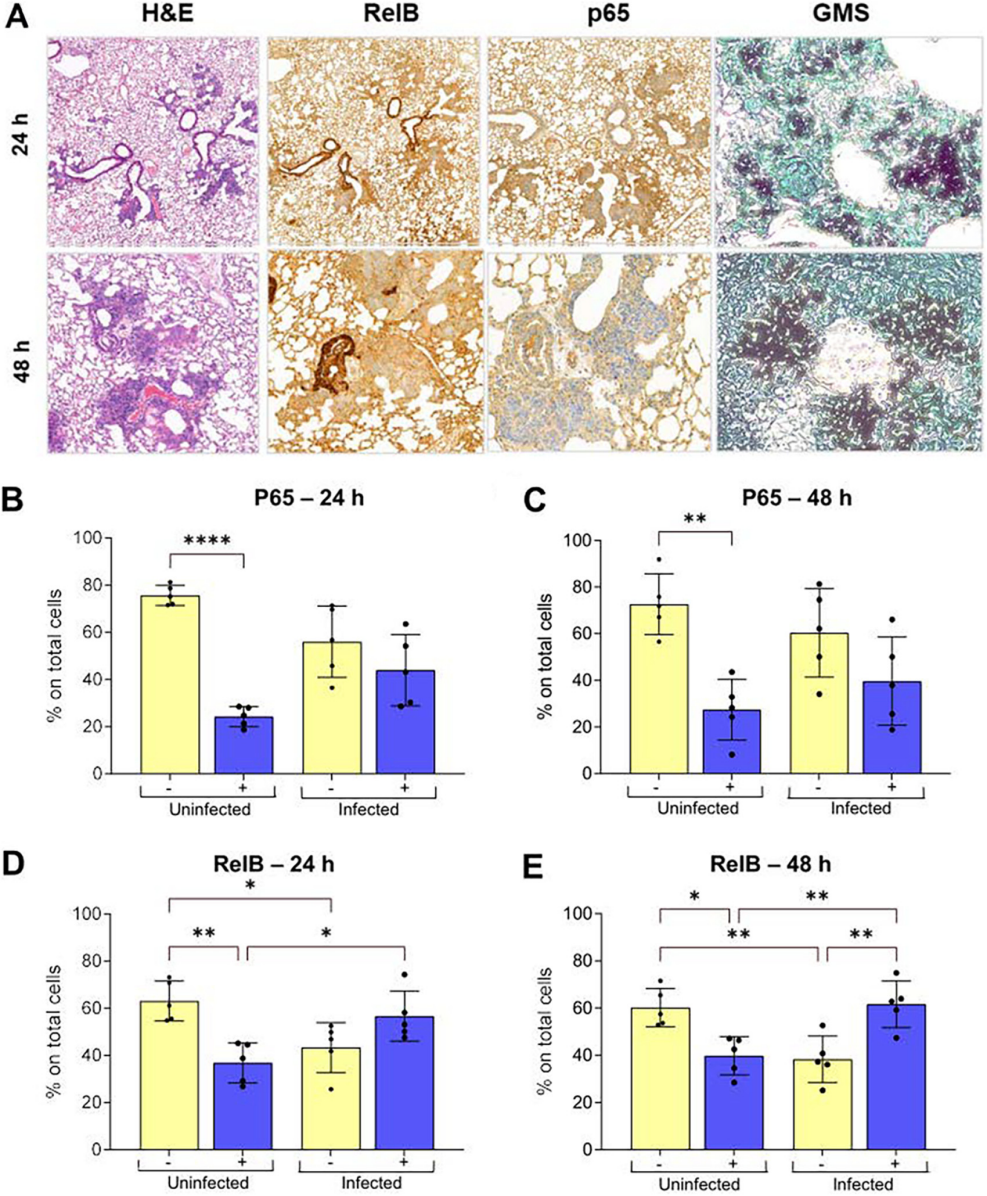
Immunohistochemistry to assess RelB and p65 immunoreactivity *in vivo* indicates subvertion of the NF-κB signaling axis following A. fumigatus infection of leukopenic mice. (A) Hematoxylin and eosin (H&E) staining, RelB/p65 immunochemistry, and GMS/green light staining of histological sections from leukopenic mice infected with A. fumigatus dTomato^ATCC46645^ for 24 and 48 h. (B to E) Quantification of p65 (B and C) and RelB (D and E) staining from scoring infected lesions and uninfected tissue in histological sections from leukopenic mice. Using the ImageJ software, nuclei were classified into 4 bins as follows: negative (intense blue), weak (light blue), moderate (light brown), and strong intensity (dark brown). Values of each category were expressed as percent relative to the total number of nuclei evaluated and reported as having positive (moderate + strong) or negative (negative + weak) immunohistochemical phenotypes. ****, *P* ≤ 0.0001; ***, *P* ≤ 0.001; **, *P* ≤ 0.01; and *, *P* ≤ 0.05.

To assess the physiological relevance of disrupted NF-κB homeostasis in an intact tissue setting, leukopenic mice were infected by intranasal instillation of A. fumigatus spores and euthanized at 24 and 48 h postinfection to examine activation of pulmonary NF-κB signaling in host tissue (Fig. 7). A semiquantitative immunohistochemical approach was applied to comparatively assess nuclear translocation of the NF-κΒ p65 and RelB subunits in the vicinity of infectious lesions (Fig. 7A). In uninfected tissues, at both 24 and 48 h postinfection, p65-negative nuclei (72 to 76%) significantly outnumbered p65-positive nuclei (Fig. 7B and C). In contrast, in infected tissues at both 24 and 48 h postinfection, equivalent numbers of p65-positive (44 to 39%) and -negative (56 to 61%) nuclei were observed (Fig. 7B and C). At both time points analyzed, positivity for RelB immunoreactivity was significantly increased in infected tissues with 57 to 61% of nuclei scoring as RelB positive in infectious lesions compared to only 37 to 40% in uninfected tissue (Fig. 7D to E). These findings substantiate the physiological relevance of our *in vitro* findings (Fig. 3E and Fig. S3E) and confirm that dysregulation of the NF-κB signaling axis is a critical component of A. fumigatus-mediated lung infection.

## DISCUSSION

**FIG 8:**
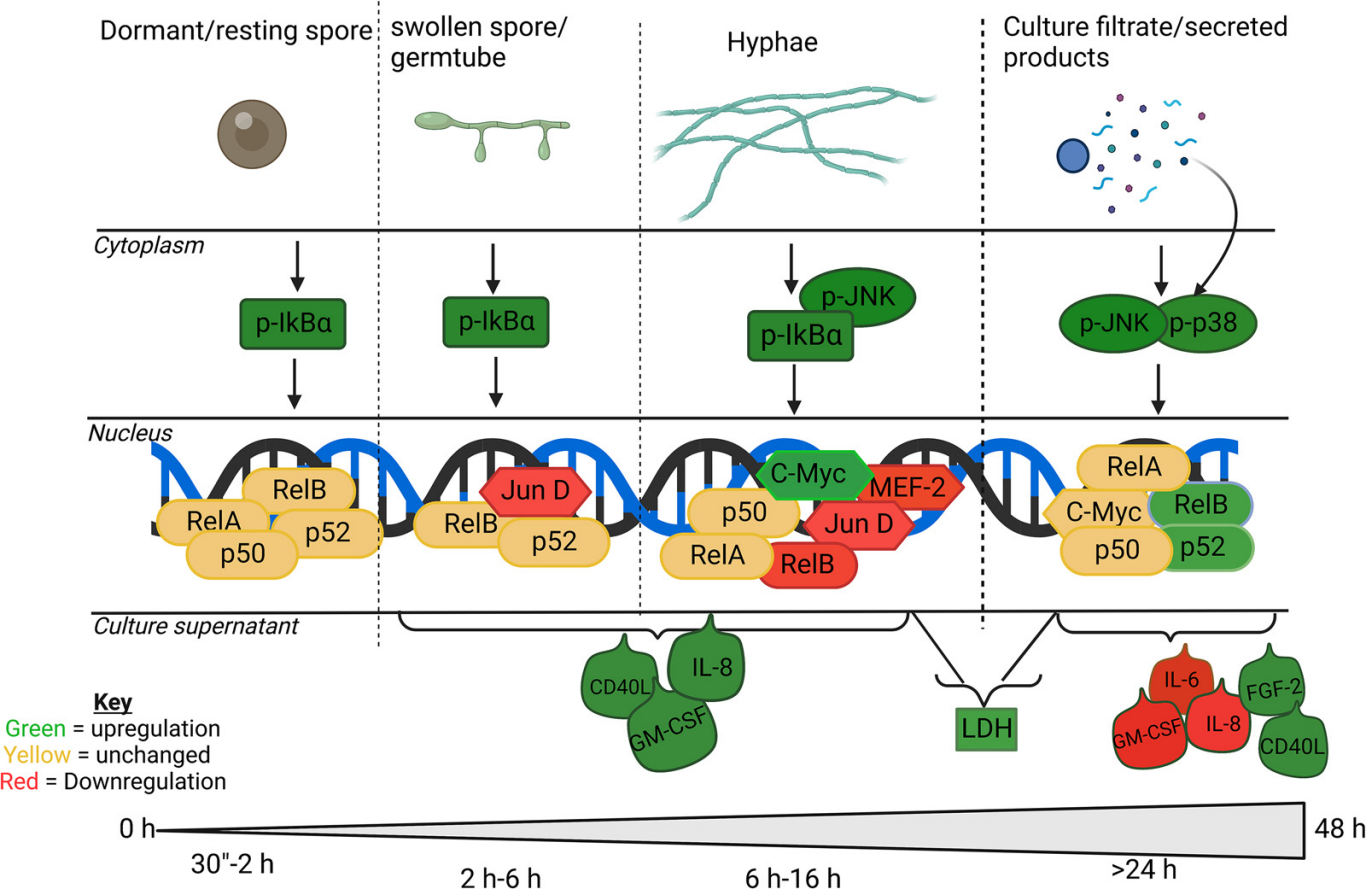
Kinetics of intracellular signaling events in alveolar type II epithelial cells in response to A. fumigatus infection. A. fumigatus conidia interact with epithelial cells activating the canonical (p50/p65) NF-κB pathway and JNK/c-Fos or c-Myc signaling specifically during hyphal growth leading to IL-8 and GM-CSF synthesis. As the fungus matures, secreted products (in the culture filtrate) interact with epithelial cells and activate 3 MAPK pathways (JNK, p38, and ERK1/2) and the noncanonical (RelB and p52) NF-κB transcription factors. This results in targeted modulation of secreted products biased toward degradation of proinflammatory mediators. Finally, cell damage measured by LDH release (cellular damage) increases as the fungal hyphae mature and secrete more soluble factors. Green and red colors indicate activation and suppression, respectively. Bold lettering indicates strong responses.

One key pathological feature of pulmonary aspergillosis is gross destruction of the lung parenchyma resulting from fungus-mediated damage; however, the precise mechanism(s) by which fungal hyphae penetrate the lining of the lung are unknown. Here, we show that the obligatory spore to hyphal morphological transition of A. fumigatus exposes epithelial cells to a wide range of cell surface-associated and secreted fungal antigens and toxins that are recognized by the host leading to the activation of multiple different host signaling pathways (Fig. 8). Previous studies showed that epithelial damage results from the combined effect of fungal secreted products (3, 28, 29) and aberrant activation of host cell signaling (17). However, the available data are sparse and fragmented and focus on single time points and morphotypes, thereby failing to account for the morphological dynamism in fungal growth that affects differential immunogenic responses. In this study, we sought to understand the AEC-Aspergillus interaction in a manner that captures the morphogenic transitions of the pathogen in a real infection by elucidating the dynamic host response to the different morphotypes of A. fumigatus and the contribution of each stage to epithelial damage. We found that airway epithelial cells respond in a distinctive and dynamic manner to A. fumigatus conidia, germlings, hyphae, and secreted fungal products via (i) immediate and sustained activation of the canonical NF-κB signaling circuit and (ii) subsequent JNK and p38 activation. Since aberrant activation of inflammatory signaling pathways can impact epithelial integrity and inhibition of such signaling has a protective effect (this work and Sharon et al. [17]), we surmise that such processes could be modulated therapeutically to protect the integrity of infected lung tissue.

This study validates our earlier findings (3) that A. fumigatus-mediated epithelial detachment occurs via at least two distinct mechanisms; first, in a contact-dependent manner, and second, by soluble hyphae-derived factors. We reveal a previously unappreciated degree of mechanistic complexity involving contact-mediated perturbation, physical invasion of the epithelial stratum by A. fumigatus germlings and hyphae, and host cell lysis via the activity of soluble effectors. Our data demonstrate cooperativity between physical fungal invasion and input from soluble effectors in achieving the overall extent of damage to the host. A similar mechanism has been demonstrated for invasive C. albicans infections whereby physical force exerted by the growing hyphae on the epithelium is complemented by specific virulence factors including the invasin Als3, several secreted proteases such as Sap5, and the peptide toxin candidalysin, which together promote degradation of tight junction proteins and barrier/membrane disruption (30–33). Our data support the hypothesis that physical invasion by A. fumigatus hyphae may be important in reducing interepithelial adhesion, thereby initiating cell detachment, whereas soluble products are required for inducing lytic cell death. We demonstrated that lytic epithelial damage is elicited via host immune responses to fungal challenge, which when ablated, using small molecule inhibitors, resulted in a significant degree of protection.

We show that cultured AECs respond in a distinctive and dynamic manner to A. fumigatus infection during the early (conidia), intermediate (germlings/immature hyphae), and late (mature hyphae/secreted effector) phases of infection, with activation of the NF-κB signaling circuit occurring within 4 h of spore exposure (Fig. 3) followed by activation of JNK signaling as spores swell and germinate (Fig. 3). As the hyphae mature, further alterations in MAPK signaling occur involving modest activation of c-*myc* and concomitant decreases in MEF2 and JunD activity (Fig. S3). Finally, in the late phase, hyphal-secreted components drive an increase in both JNK and p38 signaling (Fig. 3). These responses are similar to interactions of C. albicans and dermatophyte species with oral or skin epithelia, respectively (19). Interestingly, though, unlike *Candida* species and dermatophytes, stimulation of A549 cells with A. fumigatus did not activate p38 signaling in response to direct contact (19). Our results on NF-κB-mediated responses to conidia and germ tubes are in line with the only previous study that investigated the role of NF-κB signaling in airway Aspergillus infection; however, only a single time point of 15 h (germlings/immature hyphae) was investigated previously (15). In our study, activation of canonical NF-κB signaling occurred via a contact-dependent mechanism, since activation was absent in response to challenge with soluble hyphal products (Fig. 3). The absence of canonical NF-κB signaling following challenge with CF^48^ suggests that the ligands or effectors inducing NF-κB are not secreted proteases or soluble factors; nonetheless, the inhibition of canonical NF-κB signaling significantly reduces subsequent AEC lysis demonstrating an unfavorable outcome for host cells that subsequently mount additional responses (Fig. 4C).

Consistent with previous studies, activation of host signaling in response to early A. fumigatus infection resulted in significant increases in cytokine expression, including IL-8 and IL-6. For the first time, we report increased expression of FGF-2 (basic) release from AECs in response to early A. fumigatus infection. This finding is concordant with increased FGF-2 (basic) protein expression reported previously in the lungs of infected rats (34) and our data that demonstrate the resilience of FGF-2 (basic) to proteolytic degradation by secreted fungal proteases (Fig. 2). Exposure of AECs to CF^48^ alone leads to a significant reduction in cytokine expression and production in a concentration-dependent manner, suggesting the role of soluble factors in degrading these cytokines (35–37). The degradation in cytokines is likely mediated by proteases and possibly in a target-specific manner as some host-derived signaling factors, such as FGF-2 and CD40L, are resistant to degradation even following exposure to CF^48^ (Fig. 2). The likely physiological relevance of such observations is substantiated by a recent study that demonstrated the ability of the pathogenic yeast of Blastomyces dermatitidis to downplay host immune responses by elaborating the activities of fungal dipeptidyl-peptidase IVA (DppIVA), a close mimic of the mammalian ectopeptidase CD26 [38, 39]. DppIVA was shown to cleave human chemokines in a C-C (40) and C-X-C (41) targeted manner. Worthy of note is that the expression of the *dppIV* homolog in A. fumigatus is under PacC-mediated regulation in a murine model of invasive aspergillosis (3), a factor that might contribute to target-specific modulation of host responses and the inability of PacC null mutants to invade the respiratory epithelium.

The events identified from this study as leading to AEC decay appear to be mechanistically due to a combination of factors under the A. fumigatus master regulator PacC. Although the cohort of PacC regulated factors mediating the various damage events likely differ, it appears that fungal proteases and gliotoxin might act additively to bring about AEC lysis (Fig. 5). Interestingly, GliP and PrtT exhibit nonredundant phenotypes with respect to both JNK phosphorylation and lytic death of AECs (Fig. 5 and 6). One explanation, supported by the PacC null phenotype, is the cooperativity of gliotoxin and secreted proteases in activating host signaling. Accordingly, neither gliotoxin nor prtT null mutants are attenuated for pathogenicity in leukopenic mice (24, 26, 27, 42), but a mutant lacking both capabilities (PacC) is noninvasive and avirulent (3). We sought and tested an isogenic strain set that would allow us to compare various deficits in the expression of secreted proteases and/or gliotoxin that had been previously well-documented to reduce the cytolytic activity of A. fumigatus in *in vitro* models of infection. We have previously documented that comparison of the PacC and PrtT regulons reveals 83 and 31 secreted gene products as being downregulated in the absence of PacC or PrtT, respectively, among which only 8 genes are in common (3). It is not possible, at the current time, to confidently surmise that any or several of those 8 gene products will combine with gliotoxin to promote cytotoxicity. Indeed, as we have previously discussed, the fully invasive phenotype of the PrtT null mutant *in vivo* indicates otherwise (3).

Although this work used an *in vitro* model of alveolar carcinoma cells, the reproduction of some of the features of our *in vitro* infection model in murine infections indicates that A549 cells offer a useful alternative for recapitulating certain features of disease, including epithelial damage and NF-κB signaling. Phosphorylation-induced processing of p100 to p52 and subsequent dimerization of p52 and RelB is essential for the completion of the noncanonical NF-KB signaling responses. Indeed, a previous study showed that p100/p52 is required for RelB stabilization and the absence of p100 or its product p52 significantly reduced the level of RelB in the tissue (43). We observed an increased expression of RelB in Aspergillus-infected mouse lungs in direct correlation with inflammation and damage. A recent study reported that p52 overexpression in airway epithelial cells of transgenic mice causes increased lung inflammation, injury, and mortality following LPS stimulation (44). Another study showed that lung tissues from patients with acute respiratory distress syndrome or pulmonary adenocarcinoma exhibit increased expression of p100/p52 leading to increased cell proliferation and reduced survival (38). In keeping with our *in vitro* finding that sequential stimulation of signaling via canonical and noncanonical NF-κB pathways results from exposure of epithelia to spores and soluble fungal factors, respectively, histological analysis of infected murine tissue revealed elevated immunoreactivity of isotypic anti-p65 and anti-RelB antibodies in infected versus uninfected tissues (Fig. 7). While both canonical and noncanonical NF-κB signaling can be coordinately activated in murine alveolar epithelial cells in response to diverse stimuli (6), we do not find that RelB expression derives solely from prior activation of canonical signaling. Therefore, it is likely that among the secreted factors released by mature fungal hyphae, there exists a stimulus of RelB expression. It is not yet clear whether A. fumigatus spore- or CF^48^-induced RelB expression acts to dampen or worsen inflammatory responses and epithelial damage but it is conceivable that either scenario could disadvantage antifungal host defenses, particularly when coupled to reorchestration of inflammatory cytokine expression and cytolytic activities concomitantly expressed during hyphal invasion of the lung.

### Conclusions.

This study established the molecular and cellular basis of dynamic and temporal AEC responses to A. fumigatus infections in a tumor-derived human cell line. AECs differentially recognize the different morphotypes of Aspergillus by mounting distinct immune responses, further reinforcing the fact that the airway epithelium is functionally crucial in anti-Aspergillus immunity. Furthermore, the study demonstrated that during interaction with A. fumigatus, the AEC responses could be either destructive or protective and, in most cases, the destructive responses are driven in response to fungal secreted factors. The work presented in this study used an *in vitro* model of alveolar carcinoma cells that has proven to deliver reproducible responses to fungal challenges that can be recapitulated in the murine host. However, validation of these observations in other models, such as differentiated primary human airway epithelial cells, will be beneficial for negating artifactual effects related to dissociation and propagation of epithelial cells as two-dimensional monolayers, and/or immortalization that might introduce anomalous phenotypes. Moreover, a physiologically relevant reconstruction of the airway epithelium should approximate the appropriate three-dimensional tissue structure and multicellular composition (39).

## MATERIALS AND METHODS

### Culture of airway epithelial cells.

The A549 human alveolar type II-like carcinoma cell line (American Type Culture Collection; ATCC CCL-185) (45) was cultured in Roswell Park Memorial Institute 1640 Medium (RPMI 1640) plus glutamine, 10% fetal bovine serum (FBS), and 1% penicillin-streptomycin (10,000 units penicillin and 10 mg streptomycin/mL) at 37°C and 5% CO_2_ in a humidified atmosphere. Serum was omitted from the culture medium 18 to 24 h before fungal challenge and fungal challenges were carried out in serum-free RPMI 1640 plus glutamine and 1% penicillin-streptomycin. For transwell experiments, Calu-3 bronchial adenocarcinoma cells (ATCC HTB-55) (46) were cultured in DMEM-F12 (1×) (Thermo-Fisher Scientific) supplemented with 10% FBS and 1% penicillin-streptomycin. For chemical inhibition of signaling pathways, 1 h before fungal challenge, confluent epithelial cell monolayers were treated with the following inhibitors at concentrations previously determined (8): SP600125 (primary target: JNK1, JNK2, JNK3; 10 μM; Calbiochem), SB203580 (primary target: p38; 10 μM; Calbiochem), and BAY11-7082 (primary target: IκB-α; 2 μM; Calbiochem).

### Fungal strains and culture.

A. fumigatus isolates used in this study are listed in Data set S1. A. fumigatus was grown at 37°C for 3 to 4 days on Aspergillus complete medium (ACM) agar (47). Spores were harvested using sterile water, enumerated, and resuspended at the desired concentration in serum-free RPMI 1640 medium.

Unless otherwise stated, in *in vitro* infection assays, a final concentration of spores of 1× 10^5^ spores/mL was used for detachment and transwell experiments, while 1× 10^6^ and 1 × 1 0^7^ spores/mL were used for cytotoxicity and host signaling, respectively. To generate specific fungal morphotypes, A. fumigatus spores (10^7^ spores/mL) were pregrown in serum-free RPMI 1640 medium at 37°C with shaking at 180 RPM, for 4 h to generate swollen conidia, and for 8 or 12 h, respectively, to generate young germlings or mature hyphae. For infections with preformed fungal morphotypes, inocula were standardized by the number of spores in the inoculum and the entire biomass was used for epithelial challenge. The pregrown morphotypes were incubated for 40 min at 90°C. The killed biomass was washed by centrifugation (4,000 × *g*, 30 min, room temperature), resuspended in serum-free RPMI 1640 medium, and added to AECs monolayers.

To make CFs, freshly harvested conidia were resuspended at a cell density of 1 × 10^6^ spores/mL in RPMI 1640 medium and incubated with shaking at 200 rpm and 37°C for 16 (CF^16^) or 48 h (CF^48^). The culture suspension was filtered through sterile Miracloth (Calbiochem) and a 0.22-μm filter and then stored at −20°C until use. Unless otherwise stated, challenges of the epithelial monolayers were carried out using a 5-fold dilution of the CF^48^ in serum-free RPMI 1640 medium.

### Microscopy.

**(i) Microscopic analysis of challenged monolayers.** Upon removal of culture media and a triple wash with PBS, A. fumigatus-challenged monolayers were incubated with 1 mL of serum-free RPMI 1640 medium containing 1 μg/mL concanavalin A conjugated to FITC (Con-A; Sigma) for 30 min at 37°C and 5% CO_2_. Wide-field epifluorescence microscopy was performed using a Nikon Eclipse TE2000-E microscope (Nikon Instruments, Europe BV, UK) and a Nikon PlanFluor ×20/0.75 NA lens objective. A CoolLED PreciseExcite system (CoolLED, Andover, UK) was used with 470-nm and 550-nm LED arrays for FITC and tdTomato excitation, respectively. Images were captured with an ORCA-ER CCD camera (Hamamatsu, Welwyn Garden City, UK) driven by MetaMorph software v7.7.6.0 (Molecular Devices, Sunnyvale, CA, USA).

**(ii) Comparison of fungal biomass.** Fungal biomass of 10^4^ spores/mL of selected A. fumigatus isolates was assessed by microscopy at 4, 8, 12, 16, and 20 h of growth in supplemented RPMI 1640. Cumulative fungal length after 24 h of growth in supplemented RPMI 1640 exceeded the field of view boundaries and therefore was not measurable at the time. Wide-field epifluorescence microscopy of A. fumigatus was performed using a Nikon Ti-S microscope (Nikon Instruments, Europe BV, UK) and a Nikon PlanFluor ×20/0.75 NA lens objective. A CoolLED PreciseExcite system (CoolLED, Andover, UK) was used with a 550-nm LED array for tdTomato excitation. Images were captured with Images were captured with a Brightline LED-DA/FI/TR/CY5-A-000-ZERO multiband filter and an ORCA-ER CCD camera (Hamamatsu, Welwyn Garden City, UK) driven by Nikon Elements (v.5.11.01). A cumulative filament length (including branches) of 7 to 10 hyphae for each strain and time point was measured in FIJI using the line summation function (48).

### Analysis of A549 monolayer integrity by detachment.

Monolayers were challenged with 10^5^ A. fumigatus spores (multiplicity of infection [MOI] = 0.1) or CF. Following coincubation, monolayers were washed three times with PBS, fixed with 4% formaldehyde in PBS, and permeabilized with 0.2% Triton X-100. Nuclei of adherent A549 cells were stained with 300 nM DAPI. DAPI fluorescence in adherent epithelial cells was excited with a CoolLED PreciseExcite 380-nm LED array in combination with a Nikon UV-2A filter cube, which collected the DAPI emission. Images were acquired as stated above, where at least three fields of view, per experimental well were taken. Images were then processed and quantified using an in-house “DAPI Counter” macro written for FIJI (Data set S2). Experiments were performed in biological triplicates with three to five technical replicates, whereby a technical replicate is a different field of view for the enumeration by microscopy. Each data point in the figures represents a technical replicate of each of the biological replicates.

### Epithelial cytotoxicity.

Epithelial cytotoxicity was determined by quantification of lactate dehydrogenase activity in A549 culture supernatants at different time points post fungal challenge using the Cytox 96 NonRadioactive Cytotoxicity assay kit (Promega) according to the manufacturer's protocol and using recombinant porcine LDH enzyme (Sigma-Aldrich) for derivation of a standard curve. Experiments were performed in biological triplicates with one to four technical replicates, whereby a technical replicate is a different infection well. Each technical replicate was measured for LDH twice, and each data point in the figures represents the average of the LDH measurements for each technical replicate of each of the biological replicates.

### Measurement of transepithelial electrical resistance.

Calu-3 cells were seeded in DMEM-F12 (1×) in Transwell inserts (Scientific Laboratory Supply) placed in a 12-well tissue culture plates (Scientific Laboratory Supply) containing 2 mL of supplemented DMEM-F12. Media were changed every 2 days, and TEER was measured every 4 days using a World Precision Instrument Evom2 epithelial voltohmeter and STX-2 electrode until it reached at least 1,000 Ω·cm^−2^ (~11 to 13 days). TEER measurements were taken prior to infection with live spores or CF^48^ (TEER 0 h) and following 24 h of incubation (TEER 24 h). The decrease of TEER upon fungal challenge was calculated as the difference between TEER 24 h and TEER 0 h, normalized to fungal viable counts, and expressed as fold reduction in TEER relative to PBS challenge. Experiments were performed in biological triplicates with two technical replicates, whereby a technical replicate is a different infection well. Each data point in the figures represents a technical replicate for the biological replicates.

### Quantification of cytokine expression.

To quantify cytokine secretion, A549 cell culture supernatants were collected at 24 h postinfection with A. fumigatus spores or CF. Two distinct experimental approaches were then taken, the first adopting an unbiased semiquantitative approach based on the HXL cytokine profile kit from R&D Systems (Bio-Techne). Cell culture supernatants were diluted 1:3 and the procedure was carried out according to the manufacturer’s instructions, aquiring data using a ChemiDoc MP imaging system (Bio-Rad). The relative amount of each cytokine was calculated by deriving the mean pixel density using ImageJ and microarray profile plugins (Bio-Techne) and normalized relative to the uninfected control. To normalize between biological replicates, the data were ranked according to protein quantity and abundant proteins defined as those represented in the top or bottom quantiles were considered to be significantly up- or downregulated. In a second experimental approach, a total of 25 μL of the cell culture supernatant was used to determine cytokine levels using the luminex high-performance cytokine assay (Bio-Techne, UK) according to the manufacturer’s specifications. AECs stimulated with 50 ng/mL TNF-α served as a positive control of cytokine induction. Data were analyzed using Logistic 5PL analysis within the Bio-Plex Manager 6.1 software suite (Bio-Rad, UK).

### Quantification of phosphorylated signaling molecules in AECs.

Challenged A549 cells were lysed with modified RIPA buffer (Data set S3) and protein concentration was determined using a bicinchoninic acid (BCA) protein assay (Thermo Fisher Scientific). Phospho-p38 (p-p38), phospho-Jun N-terminal protein kinase (p-JNK), phospho-extracellular signal-regulated kinases 1 and 2 (pERK1/2), phospho-IκBα (p-IκBα), and phospho-Akt (p-Akt) were quantified in 10 μg of total protein using the Bio-Plex Pro magnetic phosphoprotein detection assay (Bio-Rad, UK). Phosphoprotein expression was normalized to β-actin and expressed as fold change relative to the uninfected (UI) control.

### Quantitation of transcription factor binding.

Nuclear proteins were isolated from challenged A549 cells using a nuclear protein extraction kit (Active Motif) according to the manufacturer’s specifications and protein concentration was determined using a BCA protein assay (Thermo Fisher Scientific). Then, 10 μg of nuclear extract was assayed, and DNA binding activities of transcription factors (ATF-2, c-Jun, FosB, Fra1, STAT-1, JunB, MEF-2, c-Myc, c-Fos, JunD, c-Jun, p50, p52, p65, RelB, and c-Rel) were assessed using the TransAM transcription factor ELISA system (Active Motif). Transcription factor binding was read at 450 nm using a Synergy 2 microplate reader (BioTek, USA) and expressed as fold change relative to the UI control.

### Murine infection.

Murine infections were performed under UK Home Office Project License PDF8402B7 in dedicated facilities at the University of Manchester. A. fumigatus spores were harvested and prepared as previously described (49) and following serial dilution, viable counts from administered inocula were determined by growth for 24 to 48 h on ACM. Mice (male CD1 outbred mice, n = 5 per time point) were rendered leukopenic by administration of cyclophosphamide (150 mg/kg, intraperitoneal) on days −3 and −1 and a single subcutaneous dose of hydrocortisone acetate (112.5 mg/kg) administered on day −1. Mice were housed in individually vented cages, anesthetized by halothane inhalation, and infected by intranasal instillation of spore suspensions with 10^8^ spores of A. fumigatus ATCC 46645. Mice were euthanized at 24 and 48 h postinfection to examine activation of pulmonary NF-κB signaling in host tissue. Immunohistochemistry was performed using the isotypic antibodies anti-p65 (ab16502; Abcam) and anti-RelB (ab180127; Abcam). Infectious lesions were identified as being (i) occluded and morphologically aberrant (ii) containing fungal hyphae. Using the Caseviewer software (3dhistech.com) the periphery of each lesion was demarcated by two boundaries, one in close apposition to the center of the lesion, and a second capturing an additional diameter of 100 pixels. The area between the two boundaries was denoted as the “near lesion region.” Each near lesion region was divided into two equal halves that were enumerated independently. Thus, for 5 infected mice, 10 data sets were generated. For the uninfected control, nuclear phenotypes were enumerated from a square section of tissue. Nuclear phenotypes were classified, by two independent observers, on the basis of intensity of nuclear staining. Using the ImageJ software, nuclei were classified into four bins as follows: negative (intense blue), weak (light blue), moderate (light brown), and strong intensity (dark brown). Values of each category were expressed as percent relative to the total number of nuclei evaluated and reported as having positive (moderate + strong) or negative (negative + weak) immunohistochemical phenotypes. Images were acquired on a 3D-Histech Panoramic-250 microscope slide-scanner using a ×20 objective (Zeiss). Snapshots of the slide scans were taken using the Case Viewer software (3D-Histech). NF-κΒ subunit (RelB and p65) immunoreactivity was assessed blindly and independently by two investigators to score negative, weak, moderate, and strong intensity.

### Statistical analysis of data.

GraphPad Prism was used to interpret data, and *P* values were calculated through unpaired t tests or one-way ordinary ANOVA tests with multiple comparison analyses as indicated. Error bars show the standard deviation (SD), with significance at ****, *P* ≤ 0.0001; ***, *P* ≤ 0.001; **, *P* ≤ 0.01; and *, *P* ≤ 0.05.
