# Supplementary material for: Aspergillus fumigatus Drives Tissue Damage via Iterative Assaults upon Mucosal Integrity and Immune Homeostasis
Source: Infect Immun. 2023 Jan 10;91(2):e00333-22. doi: 10.1128/iai.00333-22 (PMC9933693; doi:10.1128/iai.00333-22)
Supplement: Supplemental file 1 — Fig. S1 to S4 and additional supplemental material. Download iai.00333-22-s0001.pdf, PDF file, 0.3 MB [file iai.00333-22-s0001.pdf]

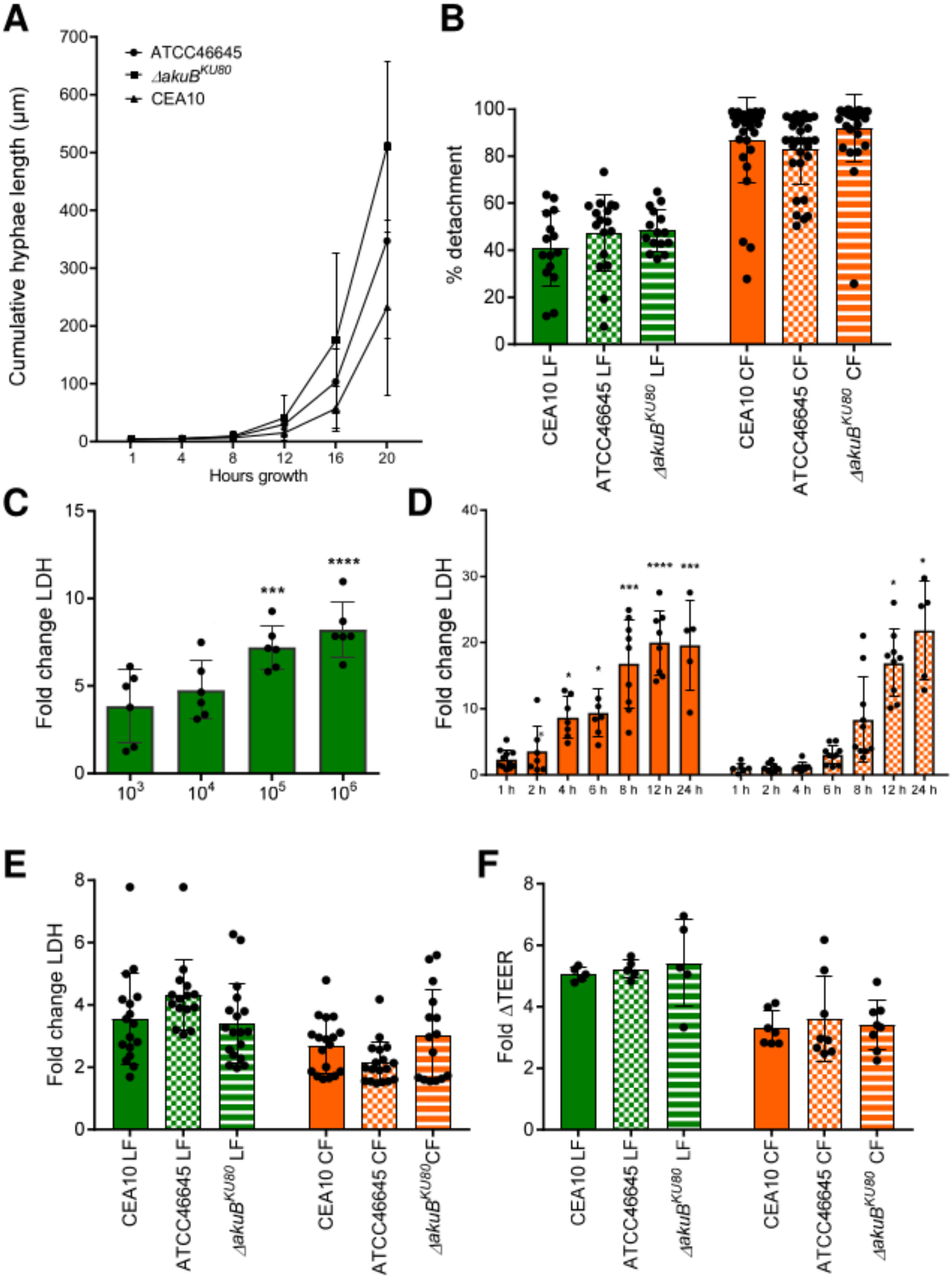
A
ATCC46645
ΔakuB^KU80
CEA10
Cumulative hyphae length (µm)
Hours growth
B
% detachment
CEA10 LF
ATCC46645 LF
ΔakuB^KU80 LF
CEA10 CF
ATCC46645 CF
ΔakuB^KU80 CF
C
Fold change LDH
***
****
D
Fold change LDH
1 h
2 h
4 h
6 h
8 h
12 h
24 h
E
Fold change LDH
F
Fold ΔTEER

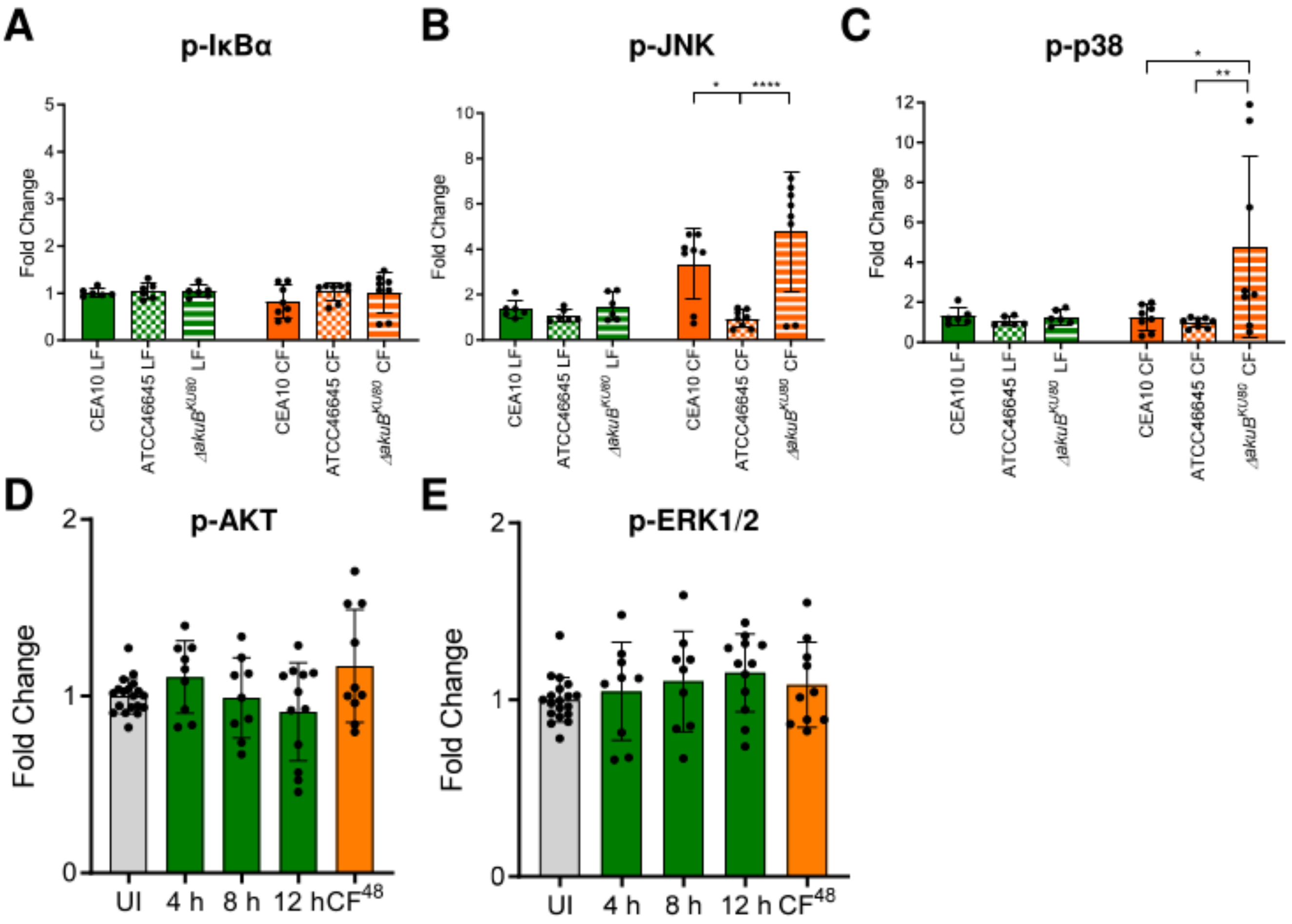

A
p-IκBα
Fold Change
CEA10 LF
ATCC46645 LF
ΔakuB^KU80 LF
CEA10 CF
ATCC46645 CF
ΔakuB^KU80 CF
B
p-JNK
C
p-p38
D
p-AKT
UI
4 h
8 h
12 h
CF^48
E
p-ERK1/2

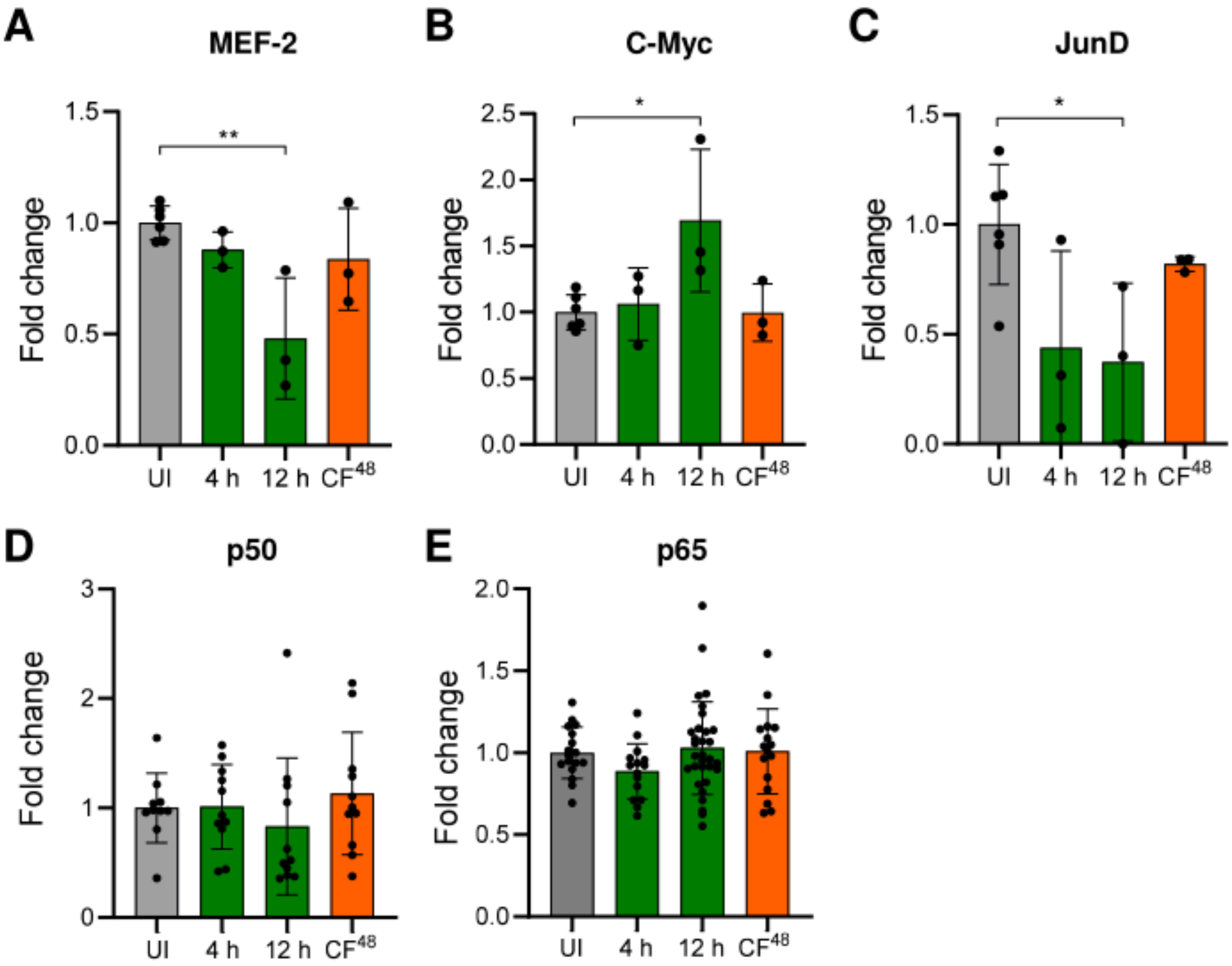
A
MEF-2
B
C-Myc
C
JunD
D
p50
E
p65
Fold change
1.5
1.0
0.5
0.0
2.5
2.0
3
2
1
0
**
*
*
UI
4 h
12 h
CF48

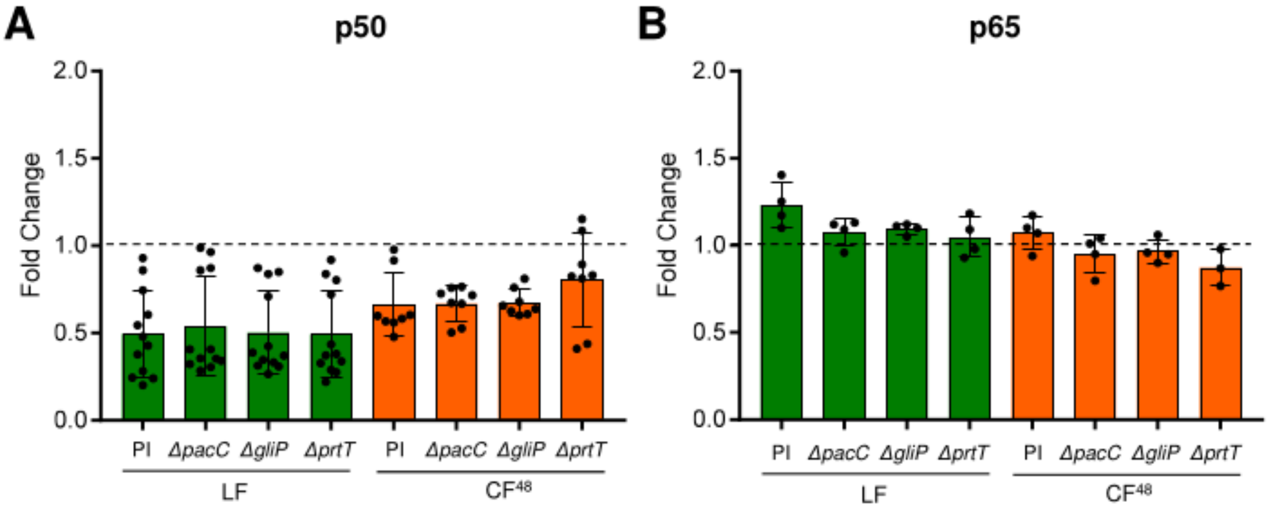

A
p50
B
p65
Fold Change
2.0
1.5
1.0
0.5
0.0
PI
ΔpacC
ΔgliP
ΔprtT
LF
CF48

Supplementary Figures

Supplementary Figure 1: Temporal quantitative analysis of epithelial decay following challenge with live *A. fumigatus* spores or CF. (A) Cumulative hyphal growth (μm) of isolates CEA10, ATCC45546 and Δaku^{Ku80} after growth at 4, 8, 12, 16 and 20 h of incubation in supplemented RPMI. **(B)** Percentage of detachment of A549 cells following infection with *A. fumigatus* CEA10, ATCC45546 and Δaku^{Ku80} spores and CF^{48} thereof. **(C)** Fold change LDH release (relative to PBS challenge) following infection with the indicated doses of *A. fumigatus* CEA10 spores for 24 h. **(D)** Fold change LDH release (relative to PBS challenge) following challenge with a 5-fold or 10-fold diluted CF^{48} from CEA10 for indicated time points. **(E)** Fold change LDH release (relative to PBS challenge) of A549 cells following infection with *A. fumigatus* CEA10, ATCC45546 and Δaku^{Ku80} spores and CF^{48} thereof. **(F)** Fold change decrease in TEER between A549 monolayers incubated for 24 and 0 h with *A. fumigatus* CEA10, ATCC45546 and Δaku^{Ku80} spores and CF^{48} thereof. Data represent the mean of three biological replicates. Error bars show ± SEM. Data were analysed by non-parametric one-way ANOVA (Kruskal-wallis test) with Dunn's multiple comparisons. Significance was calculated relative to challenge with vehicle control (PBS) unless otherwise stated. ****$p \leq 0.0001$, ***$p \leq 0.001$, **$p \leq 0.01$, and *$p \leq 0.05$

Supplementary Figure 2: Different fungal morphotypes induce differential and dynamic phosphorylation and activation of host signalling proteins in A549 cells. (A-C) Fold change (relative to uninfected control, UI) phosphorylation of NF-kB (**A**: p-IkBα) and MAPK (**B**: p-JNK and **C**: p-p38) signalling following exposure to *A. fumigatus* CEA10, ATCC45546 and Δaku^{Ku80} spores ($1x10^7$ spores/ml) for 12 h or respective 5-fold diluted CF^{48} for 4 h. **(D-E)** Fold change (relative to uninfected control, UI) phosphorylation of p-AKT **(D)** and p-ERK1/2 **(E)** following exposure to *A. fumigatus* spores ($1x10^7$ spores/ml) for indicated time points or 5-fold diluted CF^{48} for 4 h. Data represent three biological replicates with 1-5 technical replicates. Error bars show ± SEM. Data was analysed by non-parametric one-way ANOVA (Kruskal-wallis test) with Dunn's multiple comparisons. Significance was calculated relative to challenge with vehicle control (UI) and between each treatment as shown. ****$p \leq 0.0001$, ***$p \leq 0.001$, **$p \leq 0.01$, and *$p \leq 0.05$

Supplementary Figure 3: Different fungal morphotypes induce differential and dynamic activation of host transcription factors modulating epithelial host responses. (A-E) Fold change (relative to uninfected control, UI) in DNA binding activity of host transcription factors (**A**: MEF-2; **B**: c-Myc; **C**: JunD; **D**: p50 and **E**: p65) following exposure to *A. fumigatus* spores (10^7 spores/ml) for indicated time points or 5-fold diluted CF^{48} (inoculum of 10^6 spores/ml) for 4 h. Data represent three biological replicates with 1-5 technical replicates. Error bars show ± SEM. Data was analysed by non-parametric one-way ANOVA (Kruskal-wallis test) with Dunn's multiple comparisons. Significance was calculated relative to challenge with vehicle control (PBS) as shown. ****$p \leq 0.0001$, ***$p \leq 0.001$, **$p \leq 0.01$, and *$p \leq 0.05$

Supplementary Figure 4: Deletion of Δ*pacC*, Δ*gliP* and Δ*prtT* does not impact p50 and p65 signalling upon challenge with *A. fumigatus* spores or CF. Fold change (relative to uninfected control, UI) in DNA binding activity of host transcription factors (**A**: p50 and **B**: p65) following exposure to *to A. fumigatus ΔpacC, ΔgliP and ΔprtT* mutants and respective parental isolate (PI) (10^7 spores/ml) for 8 h or 5-fold diluted CF^{48} (inoculum of 10^6 spores/ml) for 4 hours. Error bars

show ± SEM. Data was analysed by non-parametric one-way ANOVA (Kruskal-wallis test) with Dunn's multiple comparisons. Significance was calculated relative to challenge with vehicle control (PBS). ****$p \leq 0.0001$, ***$p \leq 0.001$, **$p \leq 0.01$, and *$p \leq 0.05$

Supplementary Data 1

A. fumigatus **strains used in this study**

Isolate	Genotype	Reference/ Source
CEA10<br>(CBS 144-89)	Clinical isolate	[12]
ΔakuB^{KU80}	*ΔakuBku80*::*pyrGAf*-zeo	[54]
tdTomato^{ATCC46645}	[ATCC46645];*his2A*::Tdtomato	[55]
ΔpacC ^{A1160}	[A1160];*ΔakuBKu80*; *pacC::Hyg*	[56]
ΔprtT^{A1160}	[A1160];*ΔakuBKu80*;*prtT::Hyg*	[56]
ΔgliP^{CEA17}	[CEA17];*ΔakuBKU80*::*pyrGAf;gliP*::(neo-*A.n.pyrG*-neo)	[31]

Supplementary Data 2

DAPI Counter macro to automatically process and count DAPI objects

```
// DAPI Counter macro to automatically process and count DAPI objects


// This macro is optimised for wide-field imaging with a 20x 0.75NA objective lens and a 6.45um pixel size camera, giving a digital pixel size of 0.323um. If your images are of a different resolution please contact D.D.Thomson@exeter.ac.uk for assistance
// This macro was written by Darren Thomson June 2016. Any comments or quereys should be directed to  D.D.Thomson@exeter.ac.uk
```

// Instructions: Create a folder with only the fluorescent images of DAPI + create a folder to deposit the processed outlines images

// Drag and drop this macro file onto FIJI and 'Run' the script. OR create a new macro and paste this code into the window and ‘Run’ the script.

// Direct the macro to the input folder with all the DAPI images are + the output folder to deposit processed images

// Save the results data to Excel from the macro and arrange/illustrate the data accordingly in Excel.

```
dir1 = getDirectory("Where are the DAPI images");
format = getFormat();
dir2 = getDirectory("Where will I save the processed images?");
list = getFileList(dir1);
setBatchMode(true);
for (i=0; i<list.length; i++) {
 showProgress(i+1, list.length);
 open(dir1+list[i]);

run("Subtract Background...", "rolling=35");
 run("Gaussian Blur...", "sigma=3");
setAutoThreshold("Huang dark");
//run("Threshold...");
run("Convert to Mask");
run("Watershed");
run("Analyze Particles...", "size=400-5000 circularity=0.50-1.00 show=Outlines summarize");
```

```
 if (format=="8-bit TIFF" || format=="GIF")
 convertTo8Bit();
 saveAs(format, dir2+list[i]);
 close();
}
function getFormat() {
 formats = newArray("TIFF", "8-bit TIFF", "JPEG", "GIF", "PNG",
 "PGM", "BMP", "FITS", "Text Image", "ZIP", "Raw");
 Dialog.create("Batch Convert");
 Dialog.addChoice("Convert to: ", formats, "TIFF");
 Dialog.show();
 return Dialog.getChoice();
}
function convertTo8Bit() {
 if (bitDepth==24)
 run("8-bit Color", "number=256");
 else
 run("8-bit");
}
```

Supplementary Data 3

RIPA Buffer recipe

(0.05 M (50 mM) Tris-HCl pH7.5, 0.15M (150 mM) NaCl, 1% Triton X-100, 1% Sodium Deoxycholate, 0.1% SDS and 20 mM EDTA) with 10 µl/ml protease (Cat. No 78410,

Thermofisher, United Kingdom) and 10 µl/ml phosphatase (Cat. No P5726-5ML, Sigma, United Kingdom) inhibitors.

Supplementary Data 4: Cytokine profile array in response to live *A. fumigatus* and CF

Submitted as Excel spreadsheet

Human XL cytokine (HXL) profiling procedure:
To determine the global cytokine expression from epithelial cells in response to infections with live conidia and CF, cell free culture supernatant was analysed using the HXL cytokine profile kit from R&D systems according to the manufacturer's instructions. Briefly, a nitrocellulose membrane, pre-coated with same amount of capture antibody against the cytokines of interest was incubated with a blocking solution on a rocking platform for 1 h. Culture supernatant from each treatment was diluted 1:3 to a final volume of 1.5 ml. The membrane was incubated with the culture supernatant overnight at 2-8°C on a shaker. Membrane was washed 3x on a shaker for 10 min each and then incubated with detection antibody cocktail in specified diluents for 1 h. The membrane was washed as before and incubated with a 1:2000 dilution of streptavidin-phycoerythrin conjugate for 30 min. The membrane was washed and developed quickly using a 1:1 ratio of chemi-reagent 1 and 2 on a plastic sheet for 1 min at room temperature, protected from light. Excess reagent mix was removed and the membrane was covered with a plastic wrap and exposed to X-ray film for 1-10 min using a ChemoDoc MP imaging system (BIO-RAD). The relative amounts of cytokines were quantified by mean pixel density of the blots using ImageJ software and micro-array profile plugins (R &D) and normalized to the positive and negative control blots first and then to the PBS treated controls.
